# Supplementary material for: Joint contribution of socioeconomic circumstances and ethnic group to variations in preterm birth, neonatal mortality and infant mortality in England and Wales: a population-based retrospective cohort study using routine data from 2006 to 2012
Source: BMJ Open. 2019 Jul 31;9(7):e028227. doi: 10.1136/bmjopen-2018-028227 (PMC6677942; doi:10.1136/bmjopen-2018-028227)
Supplement: Supplementary data [file bmjopen-2018-028227supp001.pdf]

Supplementary material

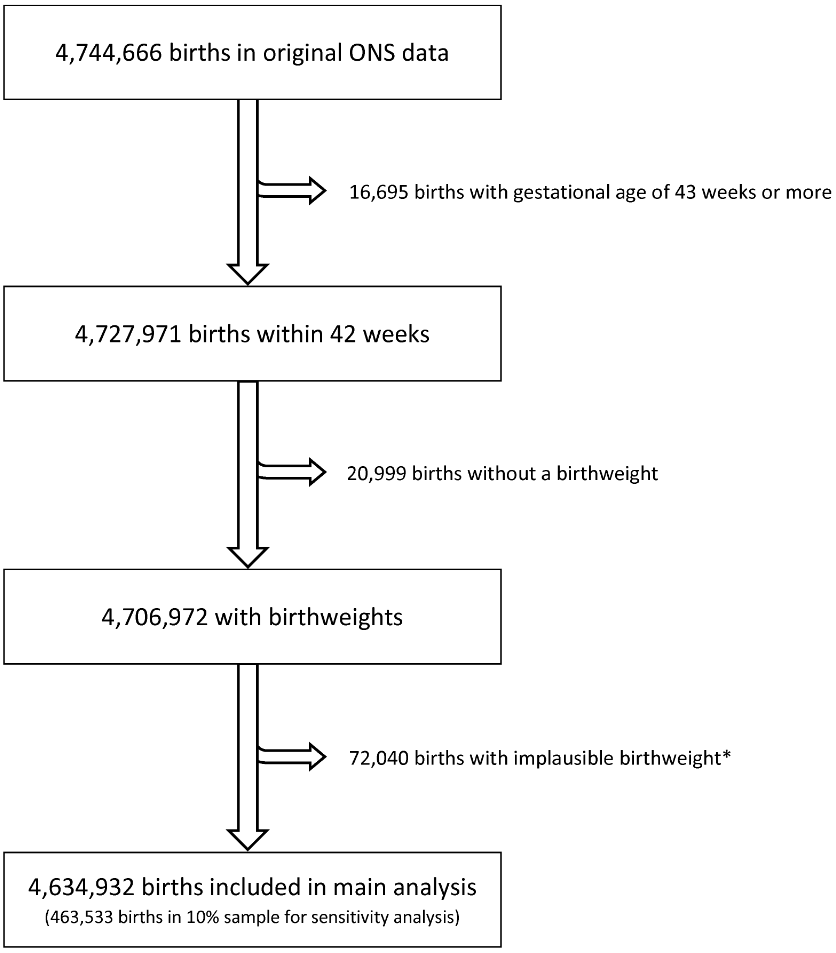

* A birthweight is considered implausible if it exceeds twice the interquartile range above and below the sex, gestation and ethnic group-specific median weight

Supplementary figure 1: Sample profile of the infants included in the analysis, England and Wales, births 2006 – 2012

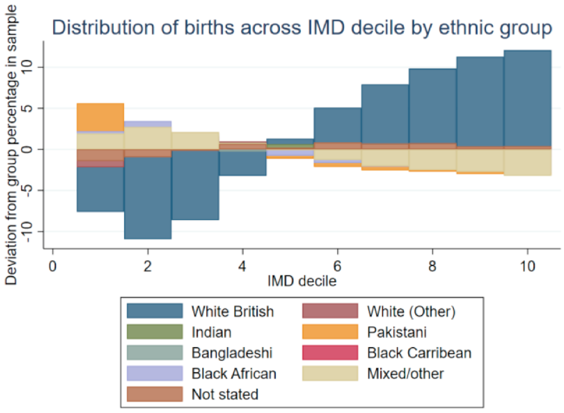

Supplementary figure 2: Distribution of births in each ethnic group and IMD relative to the ethnic group proportion of births, England and Wales, births 2006 – 2012

Opondo C, *et al. BMJ Open* 2019; 9:e028227. doi: 10.1136/bmjopen-2018-028227

Supplementary table 1: Risks of adverse birth outcomes across ethnic groups and NS-SEC classification in the 10% sample of births, England and Wales, births 2006 – 2012

	Risk of outcome		
Ethnic group	**Neonatal mortality per 1,000**	**Infant mortality per 1,000**	**Preterm birth %**
White British	1.8	2.8	5.6
White (other)	1.7	2.4	4.7
Indian	2.6	3.8	5.6
Pakistani	4.0	6.9	6.0
Bangladeshi	2.4	4.4	5.9
Black Caribbean	3.9	6.4	8.2
Black African	4.1	5.8	6.3
Mixed/other	2.3	3.7	5.7
Not stated	2.5	3.8	5.5
NS-SEC classification			
9 never worked, unemployed	3.4	5.4	7.4
8 routine occupations	2.5	4.1	6.4
7 semi-routine occupations	2.6	4.3	6.2
6 lower supervisory	2.0	3.0	5.8
5 small employers	2.0	3.5	5.5
4 intermediate occupations	2.3	3.4	5.6
3 lower managerial	1.8	2.6	5.1
2 higher professional	1.8	2.4	4.7
1 large employers	1.5	2.1	4.7
**Overall**	**2.1**	**3.3**	**5.6**

Supplementary table 2: Slope and relative indices of inequality (with bootstrap 95% confidence intervals) in the 10% sample of births, England and Wales, births 2006 – 2012

**Index of inequality across NS-SEC**	**Neonatal mortality per 1,000**	**Infant mortality per 1,000**	**Preterm birth %**
Slope index	1.41<br>(0.97 – 1.85)	2.71<br>(2.14 – 3.28)	2.33<br>(2.08 – 2.57)
Relative index	1.86<br>(1.46 – 2.26)	2.19<br>(1.78 – 2.59)	1.49<br>(1.42 – 1.56)

Opondo C, *et al. BMJ Open* 2019; 9:e028227. doi: 10.1136/bmjopen-2018-028227

Supplementary table 3: Differences in risks of adverse birth outcomes across ethnic groups, England and Wales, births 2006 – 2012

**Ethnic group**	**Difference in risk of outcome (95% CI)**					
	**Neonatal mortality per 1,000**		**Infant mortality per 1,000**		**Preterm birth %**	
	**Unadjusted**	**Adjusted**	**Unadjusted**	**Adjusted**	**Unadjusted**	**Adjusted**
White British	[comparison group]	[comparison group]	[comparison group]	[comparison group]	[comparison group]	[comparison group]
White (other)	-0.2 (-0.3 – -0.1)	-0.3 (-0.4 – -0.1)	-0.4 (-0.6 – -0.2)	-0.5 (-0.7 – -0.3)	-0.9 (-1.0 – -0.8)	-1.0 (-1.1 – -0.9)
Indian	0.5 (0.3 – 0.8)	0.4 (0.2 – 0.7)	0.7 (0.4 – 1.0)	0.6 (0.2 – 0.9)	0.5 (0.4 – 0.6)	0.3 (0.2 – 0.5)
Pakistani	2.2 (2.0 – 2.4)	1.9 (1.7 – 2.1)	4.0 (3.8 – 4.3)	3.5 (3.2 – 3.8)	0.5 (0.4 – 0.6)	-0.1 (-0.2 – 0.0)
Bangladeshi	0.9 (0.5 – 1.2)	0.5 (0.2 – 0.9)	1.5 (1.1 – 2.0)	0.9 (0.5 – 1.4)	0.8 (0.6 – 0.9)	0.2 (-0.0 – 0.3)
Black Caribbean	2.2 (1.7 –2.6)	1.9 (1.5 – 2.3)	3.1 (2.6 – 3.6)	2.6 (2.1 – 3.1)	2.7 (2.5 – 2.9)	2.2 (1.9 – 2.4)
Black African	1.5 (1.3 – 1.8)	1.2 (1.0 – 1.5)	2.3 (2.0 – 2.6)	1.8 (1.5 – 2.1)	0.6 (0.5 – 0.8)	0.1 (0.0 – 0.3)
Mixed/other	0.3 (0.2 – 0.4)	0.2 (0.0 – 0.3)	0.5 (0.4 – 0.7)	0.3 (0.1 – 0.5)	0.1 (0.0 – 0.2)	-0.1 (-0.2 – -0.1)
Not stated	0.6 (0.4 – 0.8)	0.6 (0.4 – 0.8)	0.7 (0.5 – 0.9)	0.6 (0.4 – 0.9)	0.1 (0.0 – 0.2)	0.1 (-0.0 – 0.2)

Unadjusted difference in risk of outcomes represent the total effect (path c) of ethnic group on birth outcomes; differences adjusted for socioeconomic circumstances represent the direct effect (path c')

Opondo C, *et al. BMJ Open* 2019; 9:e028227. doi: 10.1136/bmjopen-2018-028227
