# Supplementary material for: Joint contribution of socioeconomic circumstances and ethnic group to variations in preterm birth, neonatal mortality and infant mortality in England and Wales: a population-based retrospective cohort study using routine data from 2006 to 2012
Source: BMJ Open. 2019 Jul 31;9(7):e028227. doi: 10.1136/bmjopen-2018-028227 (PMC6677942; doi:10.1136/bmjopen-2018-028227)
Supplement: Author's manuscript [file bmjopen-2018-028227.draft_revisions.pdf]

BMJ Open is committed to open peer review. As part of this commitment we make the peer review history of every article we publish publicly available.

When an article is published we post the peer reviewers' comments and the authors' responses online. We also post the versions of the paper that were used during peer review. These are the versions that the peer review comments apply to.

The versions of the paper that follow are the versions that were submitted during the peer review process. They are not the versions of record or the final published versions. They should not be cited or distributed as the published version of this manuscript.

BMJ Open is an open access journal and the full, final, typeset and author-corrected version of record of the manuscript is available on our site with no access controls, subscription charges or pay-per-view fees (http://bmjopen.bmj.com).

If you have any questions on BMJ Open's open peer review process please email info.bmjopen@bmj.com

BMJ Open

The joint contribution of socioeconomic circumstances and ethnic group to variations in preterm birth, neonatal mortality and infant mortality in England and Wales – a population-based retrospective cohort study using routine data from 2006 to 2012

Journal:	*BMJ Open*
Manuscript ID	bmjopen-2018-028227
Article Type:	Research
Date Submitted by the Author:	10-Dec-2018

Complete List of Authors:	Opondo, Charles; University of Oxford, National Perinatal Epidemiology Unit<br>Gray, Ron; University of Oxford, National Perinatal Epidemiology Unit<br>Hollowell, Jennifer; University of Oxford, National Perinatal Epidemiology Unit<br>Li, Yangmei; University of Oxford, National Perinatal Epidemiology Unit<br>Kurinczuk, Jenny; University of Oxford, National Perinatal Epidemiology Unit<br>Quigley, Maria; University of Oxford, National Perinatal Epidemiology Unit

Keywords:	EPIDEMIOLOGY, PUBLIC HEALTH, PAEDIATRICS

SCHOLARONE™
Manuscripts

The joint contribution of socioeconomic circumstances and ethnic group to variations in preterm birth, neonatal mortality and infant mortality in England and Wales – a population-based retrospective cohort study using routine data from 2006 to 2012

Charles Opondo*, Ron Gray, Jennifer Hollowell, Yangmei Li, Jennifer J. Kurinczuk, Maria A. Quigley

Policy Research Unit in Maternal Health and Care, National Perinatal Epidemiology Unit, Nuffield Department of Population Health, University of Oxford, Oxford, United Kingdom

***Corresponding author's address**: Richard Doll Building, Roosevelt Drive, Headington, Oxford OX3 7LF. charles.opondo@npeu.ox.ac.uk

Word counts: abstract – 258; main text – 3,441; tables – 4; figures – 2.

Key words: socioeconomic circumstances, area deprivation, IMD, NS-SEC, ethnicity, neonatal mortality, infant mortality, preterm birth, mediation

For peer review only

For peer review only - http://bmjopen.bmj.com/site/about/guidelines.xhtml

Abstract

Objectives: This study aimed to describe the variation in risks of adverse birth outcomes across ethnic groups and socioeconomic circumstances, and to explore the evidence of mediation by socioeconomic circumstances on the effect of ethnicity on birth outcomes.

Setting: England and Wales.

Participants: The data came from the 4.6 million singleton live births between 2006 and 2012.

Exposure: The main exposure was ethnic group. Socioeconomic circumstances, the hypothesised mediator, was measured using the Index of Multiple Deprivation (IMD).

Primary and secondary outcome measures: The primary outcomes were birth outcomes, namely: neonatal death, infant death and preterm birth. We estimated the slope and relative indices of inequality to describe differences in birth outcomes across IMD, and the proportion of the variance in birth outcomes across ethnic groups attributable to IMD. We investigated mediation by IMD on birth outcomes across ethnic groups using structural equation modelling.

Results: Neonatal mortality, infant mortality and preterm birth risks were 0.2%, 0.3% and 5.6% respectively. Babies in the most deprived areas had 47% to 129% greater risk of adverse birth outcomes than those in the least deprived areas. Minority ethnic babies had 48% to 138% greater risk of adverse birth outcomes compared with white British babies. Up to a third of the variance in birth outcomes across ethnic groups was attributable to differences in IMD, and there was strong statistical evidence of an indirect effect through IMD in the effect of ethnicity on birth outcomes.

Conclusion: There is evidence that socioeconomic circumstances could be contributing to the differences in birth outcomes across ethnic groups.

For peer review only

For peer review only - http://bmjopen.bmj.com/site/about/guidelines.xhtml

Article summary

Strengths and limitations of the study

- This study is based on a large number of observations which provided sufficient statistical power to investigate associations across a variety of factors and subgroups.
- Observational data such as these are prone to unmeasured confounding; despite the reporting of adjusted estimates, some potential for residual confounding by unmeasured factors remains.

For peer review only

For peer review only - http://bmjopen.bmj.com/site/about/guidelines.xhtml

Introduction

The rates of adverse birth outcomes and indeed most other adverse health outcomes have declined steadily over the last several decades all over the world[1–3]. These trends have been attributed to several factors including improvements in access to healthcare, nutrition, sanitation and living standards, access to birth control, literacy, and advances in medical science[1,4]. In England and Wales the three and a half decades between 1970 and 2006 saw a decline in infant mortality by over 70% from 17.7 to 5.0 deaths per 1,000 live births[5]. Trends in preterm birth rates on the other hand have been more variable: although the longer-term global trend has been one of a gradual decline, over the past two decades the rates have levelled off in some parts of Europe[6,7] and even slightly increased in others[8].

Despite these mostly positive trends, stark disparities in birth outcomes persist across some individual and group characteristics. In particular, worse socioeconomic circumstances, ethnic minority status, lone parenthood, extremes of maternal age, maternal smoking, obesity and poor access and uptake of health services are all independently associated with poorer birth outcomes[9–11]. Past and recent patterns of migration in England and Wales and many other parts of Europe have resulted in increasing proportions of births to women from ethnic minority groups[12,13]. Consequently, the disproportionate burden of adverse birth outcomes among minority ethnic groups is an increasingly significant public health issue to address if we are to sustain the historical decline in most adverse birth outcomes.

Designing interventions to tackle health disparities requires an understanding of the underlying mechanisms through which heterogeneity of outcomes arise. Considering the role of ethnicity and relative deprivation, previous evidence highlights the role of ethnicity as a predictor of socioeconomic circumstances, with economic and social exclusion cited as examples of possible mechanisms of this association [14–16], and of socioeconomic circumstances as a predictor of adverse outcomes. This study therefore aimed to describe the variation in risks of adverse birth outcomes across ethnic groups and levels of socioeconomic circumstances, and to explore evidence for the role of socioeconomic circumstances in explaining ethnic variations in birth outcomes. We hypothesised that socioeconomic circumstances was a mediator of the effect of ethnic group on preterm birth and all-cause neonatal and infant mortality rates.

Methods

Study design

This was a retrospective cohort study of live births at gestational age of 22 weeks or more in England and Wales between 2006 and 2012.

For peer review only - http://bmjopen.bmj.com/site/about/guidelines.xhtml

Data sources and acquisition

Data from births and deaths registries in England and Wales have been linked since 1993. The Office for National Statistics (ONS) checks these data to minimise inconsistencies. However, these data do not include characteristics such as ethnic group and birth weight. To obtain these characteristics we requested access to data linked to the birth notifications made by midwives and birth attendants using the babies' National Health Service (NHS) numbers. Until 2015, birth notifications were done using a system called the NHS Numbers for Babies (NN4B)[17].

Data preparation

Births with missing values for gestational age and birthweight were removed from the dataset. Additionally, births with birthweight exceeding twice the interquartile range above or below the median birthweight of the sex-gestation-ethnic group-specific stratum were removed, as they were deemed implausible[18].

Outcomes, main explanatory variable and covariates

Outcomes of interest were: neonatal death, defined as a death from any cause occurring within the first 28 days of life; infant death, defined as a death from any cause occurring before the first birthday; and preterm birth, defined as a birth occurring before 37 completed weeks of gestation[19]. Gestational age in these data was intended to be based on last menstrual period, although it is likely that some were actually based on ultrasound estimates[20].

The main explanatory variable was ethnic group of the baby as recorded in the NN4B notification system, based on the list of ethnic categories used in the 2001 Census in England and Wales. The development of this list has been described elsewhere[21]. Nine categories were derived from those reported, namely White British, Other White, Indian, Pakistani, Bangladeshi, Black Caribbean, Black African, a 'Mixed/Other' group, and a 'Not stated' group[22]. These categories are broadly similar to groupings that have been used in recent studies and reports[23,24]. It is uncertain as to whether the reported ethnic group was actually the baby's and not the mother's or whether it was in fact reported by the mother and not a health professional[25].

Socioeconomic circumstances was considered a potential mediator of the association between ethnicity and birth outcomes. It was assessed using the 2015 English Index of Multiple Deprivation (IMD)[26] for

For peer review only - http://bmjopen.bmj.com/site/about/guidelines.xhtml

births in England and the 2014 Welsh IMD[27] for births in Wales. The IMD is a composite index of the relative deprivation of a defined geographical area. The index is computed from a series of indicators and statistics measuring the income, employment, health deprivation and disability, educational skills and training, housing and geographical access to services of the individuals living in an area. For the purpose of this analysis, areas were grouped into deciles of IMD, with the most deprived in the lowest decile and the least deprived in the highest decile. IMD was available for all births in the dataset.

An individual-level measure of socioeconomic circumstances, the National Statistics Socioeconomic Classification (NS-SEC), was available for a 10% sample of live births. The NS-SEC is an occupationally based measure of employment relations and conditions of occupations, conceptually relevant in showing the structure of socioeconomic circumstances and explaining various social phenomena[28]. Individuals are grouped into nine categories, which are ordered starting with employment classes corresponding to better socioeconomic circumstances. To align with the direction of ordering of IMD, NS-SEC was reverse-coded so that the lowest category corresponded to the lowest socioeconomic classification and the highest category corresponded to the highest socioeconomic classification. A household NS-SEC was derived from those of individual members of a household by assigning to the household the NS-SEC classification of the household member responsible for accommodation, or that of the member with the highest income, or that of the oldest member if incomes were equal.

The linked dataset also included covariates such as mother's age, mother's country of birth which was grouped into UK and non-UK for this analysis, birth registration type (married parents, unmarried parents living at the same address, unmarried parents living at different addresses, or a sole registrant), year of birth between 2006 and 2012, and sex of child. These variables were included in our analyses as potential confounders of the association between socioeconomic circumstances measured using IMD and birth outcomes. Mother's age and year of birth were modelled as continuous variables and any departures from linear association with the outcomes were explored and fitted.

Analysis

The slope index of inequality (SII) and the relative index of inequality (RII) were estimated to describe the differences in birth outcomes across IMD[29,30]. The SII is a measure of absolute inequality which describes gradients in outcomes across subgroups with a natural ordering. It is expressed in terms of the difference in predicted outcomes between individuals in the highest versus lowest deprivation decile. The RII is a measure of relative inequality, expressed as the ratio of predicted outcome between the highest versus lowest level of deprivation. SII and RII were estimated using the method suggested in the WHO Handbook on Health Inequality Monitoring[31], which applied regression analysis – including appropriate modelling of any departures from linearity – to predict risks of birth outcomes in the most

For peer review only - http://bmjopen.bmj.com/site/about/guidelines.xhtml

and least deprived individuals. Confidence intervals for the SII and RII were estimated by bootstrapping. As a sensitivity analysis, we repeated these analyses using household NS-SEC instead of IMD.

Two approaches were used to investigate the role of relative deprivation in the association between ethnic group and birth outcomes. First, the amount of variation in birth outcomes across ethnic groups which was explained by socioeconomic circumstances – measured using IMD deciles and modelled by linear regression – was estimated as the relative change in the residual ethnic group-level variance in outcomes as a function of relative deprivation[32,33]. The second approach, which assumed a causal link between ethnic group and IMD, and between IMD and adverse birth outcomes, involved structural equation modelling (SEM) to test an indirect effect through IMD in the effect of ethnicity on birth outcomes. This was based on the method described by Baron and Kenny[34] (Figure 1). It involved modelling the effect of ethnic group on birth outcomes (path *c*, the *total effect,* not shown in Figure 1), and of ethnic group on IMD decile (path *a*), followed by estimation of the effect of ethnic group on birth outcomes (path *c'*) when adjusted for IMD (path *b*). Since the main exposure variable was multi-categorical, the *relative* indirect effect of IMD was estimated for each ethnic group, with white British ethnic group as the comparison group [35] followed by a Sobel test to assess the strength of evidence for an indirect effect[36]. Evidence of an indirect effect in at least one group is required to conclude overall mediation for the effect of the multi-categorical exposure[35].

The relative indirect effect, i.e. the proportion of effect mediated, was calculated as *ab/c* or equivalently 1-(*c'/c*). A lack of evidence of effect in path *a* or *b* implied the absence of an indirect effect; a lack of evidence of effect in path *c* implied that there was no effect to be mediated. In either case, the relative indirect effect was meaningless, therefore not calculated. Linear, rather than logistic, regression was preferred for modelling all effects despite the birth outcomes being binary variables because explained variance measures in linear models are most intuitive,[37,38] as is the causal decomposition of bivariate associations in linear models, and given that the large number of observations in this dataset, the coefficients and standard errors from linear regression of binary outcomes are expected to be identical to logistic regression[39]. To correct for multiple testing, we used a stringent threshold of $p<0.001$ when assessing the evidence for direct and indirect effects. Goodness-of-fit of the SEM was tested using the root mean square error of approximation (RMSEA)[40], the comparative fit index (CFI)[41] and the Tucker-Lewis index (TLI)[42]. These indices range in value between 0 and 1. Smaller values of the RMSEA and larger values of both CFI and TLI represent better fit.

Data management, manipulation and analyses were performed using Stata v13[43].

For peer review only - http://bmjopen.bmj.com/site/about/guidelines.xhtml

Patient and public involvement

A Patient and Public Involvement (PPI) group was formed to advise on the analysis plan, interpretation of results, and dissemination plans for this study. In particular, the group provided guidance on ethnic groupings that were used in this study.

Results

Data on 4,744,666 infants were available in the linked dataset. We excluded 72,040 infants with implausible birthweights. A further 20,999 infants with missing birthweights and 16,695 with gestational age of 43 weeks and above, were excluded. A total of 4,634,932 infants were included in the main analysis and 463,533 in the sensitivity analysis (supplementary figure 1).

Table 1 is a summary of the characteristics of the infants included in the main analysis. The infants were largely of white British ethnic origin (65%) and mostly born to UK-born mothers (76%). The proportion of births in which the child's ethnicity was not stated was 6.2% overall, although it decreased from 9.9% in 2006 to 3.3% in 2012. There were 2.6% more male than female infants in the dataset. The number of births was distributed almost evenly over the 7 years covered by the dataset, although there was a gradual year-on-year increase in the number of births. The mean age of mothers was 29 years. Over half of registrations were by married parents and almost a third were by unmarried cohabiting parents. The rest were either joint registrations by parents living separately or single-parent registrants.

The overall proportion of preterm births was 5.6%. Infant and neonatal deaths were less common, occurring in 3.24 and 2.08 per 1,000 live births, respectively. However, more adverse birth outcomes occurred in infants born in deprived areas, overall (Table 2) and within ethnic group (Figure 2), and a disproportionate number of adverse outcomes occurred among ethnic minority infants (Table 2). Additionally, there were disproportionately more ethnic minority infants born in socio-economically deprived areas. In particular, there were higher proportions of black, Pakistani and Bangladeshi infants in the lower IMD deciles than the overall proportion of infants of these ethnicities, but a lower proportion of the same ethnicities in the higher IMD deciles; the opposite was true for white infants (Supplementary figure 2). The distribution of risks of adverse outcomes across ethnic groups in the 10% sample used for the sensitivity analysis was virtually identical to that in the overall sample (Supplementary table 1).

The slope and relative indices of inequality across birth outcomes are presented in Table 3. They show that infants born in the most socio-economically deprived areas had between 1.47 and 2.29 times the risk of adverse birth outcomes of those in the least deprived areas. The slope and relative indices of adverse birth outcomes across household NS-SEC were similar to those based on IMD: infants born in

For peer review only - http://bmjopen.bmj.com/site/about/guidelines.xhtml

the most socio-economically deprived households had between 1.49 and 2.19 times the risk of adverse birth outcomes of those in the least deprived households (Supplementary table 2). Table 3 also shows that a small proportion, between 0.7% and 3.1%, of the overall variance in birth outcomes at ethnic group level was attributable to within-group differences. This implies that most of the variance in birth outcomes was due to between-group differences. Between 25.8% and 35.1% of the variance in outcomes at ethnic group level was explained by IMD in both crude and adjusted regression models.

There was strong evidence of an indirect effect of ethnic group on birth outcomes through socioeconomic circumstances (Sobel test p-values <0.001). The relative indirect effects varied across ethnic groups and across outcomes, from -20.1% for infant mortality in white non-British infants to 78.2% for preterm birth in Black African infants, all relative to white British infants (Table 4). Given that the indirect effects were estimated relative to the white British group, negative indirect effect did not imply suppression as it would in absolute mediation models, but a lower indirect effect than the comparison group. The structural equation models were a good fit for the data: in all models the RMSEA was <0.001 and both CFI and TLI were >0.999.

For peer review only

Table 1: Characteristics of the 4,634,932 infants born in England and Wales, 2006 – 2012

Ethnic group, n (%)	
White British	3,009,231 (64.9)
White (other)	340,526 (7.4)
Indian	132,651 (2.9)
Pakistani	180,269 (3.9)
Bangladeshi	62,948 (1.4)
Black Caribbean	47,505 (1.0)
Black African	154,076 (3.3)
Mixed or other ethnic group	419,970 (9.1)
Ethnic group not stated	287,756 (6.2)
Sex of infant, n (%)	
Male	2,377,766 (51.3)
Female	2,257,166 (48.7)
Year of birth, n (%)	
2006	631,705 (13.6)
2007	646,902 (14.0)
2008	663,918 (14.3)
2009	659,807 (14.2)
2010	671,265 (14.5)
2011	675,075 (14.6)
2012	686,260 (14.8)
Mother's age in years, mean (SD)	29.0 (6.0)
Mother's country of birth*, n (%)	
UK	3,507,324 (75.7)
Not UK	1,127,465 (24.3)

For peer review only - http://bmjopen.bmj.com/site/about/guidelines.xhtml

Birth registration type, n (%)	
Registration by married parents	2,499,063 (53.9)
Joint registration by unmarried parents (same address)	1,398,935 (30.2)
Joint registration by unmarried parents (different addresses)	450,500 (9.7)
Sole registrant	286,434 (6.2)
IMD decile, n (%)	
1 (most deprived)	662,767 (14.3)
2	598,259 (12.9)
3	541,795 (11.7)
4	489,932 (10.6)
5	439,270 (9.5)
6	422,908 (9.1)
7	388,665 (8.4)
8	382,585 (8.3)
9	366,851 (7.9)
10 (least deprived)	341,900 (7.4)
Preterm birth (<37 weeks gestation), n (%)	258,515 (5.6)
Neonatal mortality, n (%)	9,638 (0.2)
Infant mortality, n (%)	15,001 (0.3)
*Excludes 143 infants whose mother's country of birth is unclear or not stated	

Table 2: Risks of adverse birth outcomes across ethnic groups and socioeconomic circumstances, England and Wales, births 2006 – 2012

	**Risk of outcome per 1,000 live births**		
Ethnic group	**Neonatal mortality**	**Infant mortality**	**Preterm birth**
White British	1.8	2.9	55.4
White (other)	1.6	2.5	46.2
Indian	2.4	3.6	60.2
Pakistani	4.0	6.9	60.0
Bangladeshi	2.7	4.4	63.0
Black Caribbean	4.0	6.0	82.1
Black African	3.4	5.2	61.9
Mixed/other	2.1	3.4	56.4
Not stated	2.4	3.5	54.4
Index of multiple deprivation decile			
1 – most deprived	2.8	4.7	67.0
2	2.6	4.1	62.9
3	2.5	3.8	59.9
4	2.1	3.3	56.8
5	1.9	2.9	53.4
6	1.8	2.7	51.7
7	1.6	2.4	50.4
8	1.6	2.3	48.5
9	1.5	2.2	47.5
10 – least deprived	1.5	2.1	45.0
**Overall**	**2.1**	**3.2**	**55.8**

For peer review only - http://bmjopen.bmj.com/site/about/guidelines.xhtml

Table 3: Slope and relative indices of inequality (bootstrap 95% confidence intervals), and amount of variation in birth outcomes across ethnic groups explained by IMD, England and Wales, births 2006 – 2012

	Neonatal mortality	Infant mortality	Preterm birth
**Index of inequality across IMD**			
Slope index	1.48 (1.35 – 1.60)	2.66 (2.50 – 2.82)	21.54 (20.95 – 22.14)
Relative index	2.05 (1.92 – 2.18)	2.29 (2.17 – 2.41)	1.47 (1.46 – 1.49)
**Proportion of total variance:**			
within ethnic group (crude)	2.7%	3.1%	0.7%
explained by IMD (crude)	30.1%	34.0%	28.2%
explained by IMD (adjusted*)	25.8%	27.3%	35.1%

*adjusting for mother's age, mother's country of birth, registration type, year of birth and sex of child

Table 4: Relative indirect effect of ethnic group on birth outcomes, with 95% confidence intervals, England and Wales, births 2006 – 2012

	Neonatal mortality	Infant mortality	Preterm birth
White British	[comparison group]	[comparison group]	[comparison group]
White (other)	_	-20.1% (-35.9 to -4.4)	-8.8% (-9.5 to -8.0)
Indian	14.7% (8.5 to 20.1)	21.0% (10.4 to 31.6)	29.7% (19.9 to 39.4)
Pakistani	14.0% (11.3 to 16.7)	13.9% (12.2 to 15.6)	_
Bangladeshi	_	40.0% (24.4 to 55.2)	_
Black Caribbean	13.4% (10.5 to 16.3)	16.8% (13.0 to 20.7)	19.3% (17.0 to 21.5)
Black African	18.6% (14.1 to 23.1)	22.6% (18.5 to 27.0)	78.2% (64.2 to 92.2)
Mixed or other	_	_	_
Not stated	1.6% (0.8 to 2.3)	2.5% (0.6 to 4.4)	_

Indirect effect was not calculated in groups and outcomes where there was no evidence of a direct or total effect

Discussion

These data from a cohort of babies born in England and Wales between 2006 and 2012 showed poorer birth outcomes among infants of non-white minority ethnic groups. Specifically, ethnic minority infants born over this period had up to twice the risk of adverse birth outcomes of white British infants. These data also showed that among all ethnic groups there was evidence of a gradient in birth outcomes across levels of socioeconomic circumstances, with infants born in the most deprived areas experiencing between 1.47 and 2.29 times the risk of adverse birth outcomes compared with those born in the least deprived areas. Additionally, minority ethnic infants were more likely to be born in deprived areas. Our study aimed to explore the evidence for relative deprivation as the driving mechanism for differences in birth outcomes across ethnic groups. This was based on a causal model in which we hypothesised that relative deprivation was a consequence of minority ethnic status, which was then a cause of poorer birth outcomes.

For peer review only - http://bmjopen.bmj.com/site/about/guidelines.xhtml

We found that between a quarter and a third of the total between-ethnic group variance in birth outcomes was explained by relative deprivation. This finding, along with the evidence of the association between ethnicity and relative deprivation, and between relative deprivation and adverse birth outcomes, is consistent with a causal link between these factors, although not sufficient proof of causality. We also found strong evidence of an indirect effect through relative deprivation in the association between ethnic group and birth outcomes, with between 13.4% and 78.2% of the risk of adverse birth outcomes in non-white minority ethnic group infants estimated to be due to the higher levels of deprivation in these groups when compared to white British infants. Compared to white non-British infants relative deprivation mediated 8.8% to 20.1% of the excess risk of adverse birth outcomes in white British infants. Together, these findings support the hypothesis that relative deprivation is one mediating mechanism through which observed differences in birth outcomes across ethnic groups arise; there may be other mechanisms, beyond the scope of this study, suggested by the finding of incomplete mediation across all groups and outcomes.

These conclusions are further supported by previous studies which highlight the associations between ethnicity, relative deprivation and birth outcomes. For example, other studies have shown that health outcomes in infants and children tend to worsen with greater area-level poverty[44], lower household income or in lower social classes[45], all of which are indicators of relative deprivation. This pattern is also repeated in adults: overall life expectancy for men and women living in the most deprived regions of England were 8.2 and 6.9 years, respectively, lower than those living in the least deprived regions in 2013[46]. Furthermore, previous studies[16,47] and these data also show a greater propensity for minority ethnic groups to live in socio-economically deprived areas and to suffer poorer outcomes. However, none of the studies we reviewed investigated the potential mediating effect of socioeconomic circumstances in the association between ethnicity and birth outcomes.

We have also assumed that the relationship between ethnicity and socioeconomic circumstances, and between socioeconomic circumstances and adverse health outcomes are causal. A number of established findings support these assumptions of causality and highlight possible mechanisms of effect. For example, language and cultural differences between minority and majority groups can create barriers to accessing or benefiting from information[48]. Minority groups are also more likely to experience various forms of discrimination based on their race, religion or culture, all of which may systematically disadvantage them in accessing services, housing and credit[49]; gaining employment[50], highlighted by the employment gap[51], and rising up the ranks while in employment[52]. In turn, poor socioeconomic circumstances can lead to poor health outcomes through: behavioural mechanisms e.g. tobacco, drug and alcohol use, poorer diet, lower utilisation of health services and contraception[10,53]; reduced access to material resources and safe environments resulting in greater mortality from pollution, poor housing and other health hazards; greater life stress caused by personal circumstances, leading to greater risk of

For peer review only - http://bmjopen.bmj.com/site/about/guidelines.xhtml

depression, anxiety and poor mental health; with the cumulative effect of all these potential mechanisms over the life course on health and wellbeing within and across generations[54].

One limitation of this study is in the use of observational data to explore the causal relationships implied by the mediation model. Observational data are more prone to the occurrence of unmeasured confounding or reverse causality between the main exposure and the outcome, the mediator and the outcome, and between the exposure and the mediator. However, given that the exposure in this study was ethnic group, there were unlikely to be any common causes or reverse causality between it and other factors. And although there was unlikely to be any reverse causality between relative deprivation and birth outcomes, this association was likely to be confounded by unmeasured factors since both factors may have had external common predictors. In our analysis, we adjusted this pathway for mother's age, mother's country of birth, birth registration type (a proxy measure for parental marital status), year of birth and sex of the child. Although this adjustment did not include factors such as education which may be common predictors of both relative deprivation and birth outcomes[55], the definition of IMD is broad and encompasses some of these factors, about which we did not have information in this dataset – a further limitation of the explanatory power of the study. We also did not have information about important parental factors such as smoking; therefore, there remains some potential for residual confounding. Another limitation of this study is the use of area level deprivation to model the socioeconomic circumstances of individuals; this was necessitated by the fact that individual level indicators of socioeconomic circumstances were not available for the whole sample. This has partly been addressed in the sensitivity analysis in which we have used family NS-SEC derived from parental NS-SEC as an alternative approach to measuring socioeconomic circumstances.

These findings suggest that with increasing ethnic diversity in England and Wales, further reductions in adverse birth outcomes could be facilitated by interventions that reduce overall social inequality as this appears to be an important driver of variation in outcomes across ethnic groups. Such interventions must broaden their focus beyond health outcomes alone, and must aim to improve the totality of life circumstances among groups who experience the poorest outcomes.

For peer review only

For peer review only - http://bmjopen.bmj.com/site/about/guidelines.xhtml

Acknowledgements

We thank Bliss, the Race Equality Foundation, Sands, and the Lullaby Trust for their helpful involvement in the Patient and Public Involvement (PPI) consultation for this project. We also thank Alison MacFarlane, Hiranthi Jayaweera and Nirupa Dattani for their input during the planning and writing up of this study.

Author contributions

RG, JH, JJK, and MAQ conceived the study and were responsible for data acquisition. CO performed the statistical analysis with support from RG and MAQ. CO wrote the first draft, to which RG, JH, YL, JJK, and MAQ contributed and provided feedback during its development. All authors read and approved the final version of the manuscript.

Competing interests

All authors have completed the ICMJE uniform disclosure form at http://www.icmje.org/coi_disclosure.pdf and declare: no support from any organisation for the submitted work; no financial relationships with any organisations that might have an interest in the submitted work in the previous three years, no other relationships or activities that could appear to have influenced the submitted work.

Data availability

The data were provided by the Office for National Statistics (ONS) under a contractual agreement that does not permit the sharing of data. All requests for data access should be made directly to the ONS.

Ethical approval

The project was approved by the NRES Committee South Central - Oxford B, Ref 15/SC/0493.

Funding

This paper reports on an independent study which was funded by the NIHR Policy Research Programme in the Department of Health and Social Care, England (grant 108/0001). The Department of Health was

For peer review only - http://bmjopen.bmj.com/site/about/guidelines.xhtml

not involved in any aspects of the study, and the views expressed are not necessarily those of the Department.

References

1. World Health Organization. Health across the life span. *World Heal Rep 1998*. 1998:61-111. http://www.who.int/whr/1998/en/whr98_ch3.pdf. Accessed March 8, 2018.

2. Black RE, Cousens S, Johnson HL, et al. Global, regional, and national causes of child mortality in 2008: a systematic analysis. *Lancet*. 2010;375(9730):1969-1987. doi:10.1016/S0140-6736(10)60549-1

3. Liu L, Johnson HL, Cousens S, et al. Global, regional, and national causes of child mortality: An updated systematic analysis for 2010 with time trends since 2000. *Lancet*. 2012;379:2151-2161.

4. Clift J, International Monetary Fund. *Health and Development : A Compilation of Articles from Finance & Development*. (Clift J, ed.). Washington; 2004. https://www.imf.org/external/pubs/ft/health/eng/hdwi/hdwi.pdf. Accessed March 8, 2018.

5. Norman P, Gregory I, Dorling D, Baker A. Geographical trends in infant mortality: England and Wales, 1970-2006. *Health Stat Q*. 2008;(40):18-29. https://search.proquest.com/docview/232834485/fulltextPDF/2F64288B1A974531PQ/1?accountid=13042. Accessed March 8, 2018.

6. Jakobsson M, Gissler M, Paavonen J, Tapper AM. The incidence of preterm deliveries decreases in Finland. *BJOG An Int J Obstet Gynaecol*. 2008;115(1):38-43. doi:10.1111/j.1471-0528.2007.01565.x

7. Schaaf J, Mol B, Abu-Hanna A, Ravelli A. Trends in preterm birth: singleton and multiple pregnancies in the Netherlands, 2000-2007. *BJOG An Int J Obstet Gynaecol*. 2011;118(10):1196-1204. doi:10.1111/j.1471-0528.2011.03010.x

8. Zeitlin J, Szamotulska K, Drewniak N, et al. Preterm birth time trends in Europe: A study of 19 countries. *BJOG An Int J Obstet Gynaecol*. 2013;120(11):1356-1365. doi:10.1111/1471-0528.12281

9. Office for National Statistics. *Pregnancy and Ethnic Factors Influencing Births and Infant Mortality: 2013*. London; 2015. https://www.ons.gov.uk/peoplepopulationandcommunity/healthandsocialcare/causesofdeath/bulletins/pregnancyandethnicfactorsinfluencingbirthsandinfantmortality/2015-10-14.

10. World Health Organisation. *Social Determinants of Health*. Second Edi. (Wilkinson R, Marmot M, eds.). Copenhagen; 2003.

11. The Marmot review. *The Marmot Review: Fair Society, Healthy Lives*. London; 2010. http://www.instituteofhealthequity.org/resources-reports/fair-society-healthy-lives-the-marmot-review/fair-society-healthy-lives-full-report-pdf.pdf. Accessed March 9, 2018.

12. Cangiano A. Net Migration as a Target for Migration Policies: A Review and Appraisal of the UK Experience. *Int Migr*. 2016;54(2):18-33. doi:10.1111/imig.12223

13. Coleman D. Immigration , Population and Ethnicity: The UK in International Perspective. *Migr Obs*. 2013:1-11. www.migrationobservatory.ox.ac.uk. Accessed March 13, 2018.

14. Nazroo JY. The structuring of ethnic inequalities in health: Economic position, racial

For peer review only - http://bmjopen.bmj.com/site/about/guidelines.xhtml

discrimination, and racism. *Am J Public Health*. 2003;93(2):277-284. doi:10.2105/AJPH.93.2.277

15. Karlsen S, Nazroo JY. Relation between racial discrimination, social class, and health among ethnic minority groups. *Am J Public Health*. 2002;92(4):624-631. doi:10.2105/AJPH.92.4.624

16. Nazroo J, Karlsen S. Ethnic inequalities in health: social class, racism and identity. *Res Find*. 2001;10(10). http://www.lancaster.ac.uk/fass/projects/hvp/pdf/fd10.pdf. Accessed December 9, 2017.

17. Hilder L, Moser K, Dattani N, Macfarlane A. Pilot linkage of NHS Numbers for Babies data with birth registrations. *Heal Stat Q*. 2007;(33):25-33. https://pdfs.semanticscholar.org/128c/a2310d5d5028d5a5cc9a69d27dc3ac20f385.pdf. Accessed March 13, 2018.

18. Tukey J. *Exploratory Data Analysis*. Addison-Wesley; 1977. https://pdfs.semanticscholar.org/2f40/fd06bc9fd00a27437e14ed171e96e4fd9326.pdf. Accessed February 1, 2018.

19. World Health Organization. *International Statistical Classification of Diseases and Related Health Problems - 10th Revision (ICD10)*. WHO; 2010. http://apps.who.int/classifications/icd10/browse/Content/statichtml/ICD10Volume2_en_2010.pdf.

20. Moser K, Hilder L. Assessing quality of NHS Numbers for Babies data and providing gestational age statistics. *Health Stat Q*. 2008;37:15-23. http://researchonline.lshtm.ac.uk/24366/.

21. Office for National Statistics. *Ethnic Group Statistics: A Guide for the Collection and Classification of Ethnicity Data*. London; 2003. http://scholar.google.com/scholar?hl=en&btnG=Search&q=intitle:Ethnic+group+statistics+A+guide+for+the+collection+and+classification+of+ethnicity+data#1.

22. Li Y, Quigley MA, Dattani N, et al. The contribution of gestational age, area deprivation and mother's country of birth to ethnic variations in infant mortality in England and Wales: A national cohort study using routinely collected data. *PLoS One*. 2018;13(4):e0195146. doi:10.1371/journal.pone.0195146

23. Kurinczuk JJ, Hollowell J, Brocklehurst P, Gray R. *Inequalities in Infant Mortality Project Briefing Paper 1. Infant Mortality: Overview and Context*. Oxford; 2009. https://www.npeu.ox.ac.uk/downloads/files/infant-mortality/Infant-Mortality-Briefing-Paper-1.pdf.

24. Bosveld K, Connolly H, Rendall M. *A Guide to Comparing 1991 and 2001 Census Ethnic Group Data.* London; 2006. http://webarchive.nationalarchives.gov.uk/20151014015820/http://www.ons.gov.uk/ons/rel/ethnicity/focus-on-ethnicity-and-identity/a-guide-to-comparing-1991-and-2001-census-ethnic-group-data/comparing-1991-and-2001-census-ethnic-group-data.pdf.

25. Moser K, Stanfield K, Leon D. Birthweight and gestational age by ethnic group, England and Wales 2005: introducing new data on births. *Health Stat Q*. 2008;(39):22-31, 34-55. http://www.ncbi.nlm.nih.gov/pubmed/18810886. Accessed November 27, 2017.

26. Smith T, Noble M, Noble S, Wright G, McLennan D, Plunkett E. *The English Indices of Deprivation 2015 - Technical Report.*; 2015. doi:http://dx.doi.org/http://www.communities.gov.uk/publications/corporate/statistics/indices2010technicalreport

27. Welsh Government. Welsh Index of Multiple Deprivation (WIMD) 2014. *Off Natl Stat*. 2014:104. doi:10.1136/vr.h476

For peer review only

For peer review only - http://bmjopen.bmj.com/site/about/guidelines.xhtml

28. Office for National Statistics. The National Statistics Socio-Economic Classification. https://www.ons.gov.uk/methodology/classificationsandstandards/otherclassifications/thenationalstatisticssocioeconomicclassificationnssecrebasedonsoc2010. Published 2016. Accessed June 14, 2018.

29. Regidor E. Measures of health inequalities: part 2. *J Epidemiol Community Heal*. 2004;58(11):900-903. doi:10.1136/jech.2004.023036

30. Pamuk ER. Social-class inequality in infant mortality in England and Wales from 1921 to 1980. *Eur J Popul*. 1988;4(1):1-21. doi:10.1007/BF01797104

31. WHO. Handbook on health inequality monitoring: with a special focus on low- and middle-income countries. http://apps.who.int/iris/bitstream/10665/85345/1/9789241548632_eng.pdf. Accessed March 13, 2017.

32. LaHuis DM, Hartman MJ, Hakoyama S, Clark PC. Explained variance measures for multilevel models. *Organ Res Methods*. 2014;17(4):433-451. doi:10.1177/1094428114541701

33. Snijders T, Bosker R. Modeled variance in two-level models. *Sociol Methods Res*. 1994;22(3):342-363. doi:10.1177/0049124194022003004

34. Baron RM, Kenny DA. The moderator-mediator variable distinction in social psychological research: conceptual, strategic, and statistical considerations. *J Pers Soc Psychol*. 1986;51(6):1173-1182. doi:10.1037/0022-3514.51.6.1173

35. Hayes AF, Preacher KJ. Statistical mediation analysis with a multicategorical independent variable. *Br J Math Stat Psychol*. 2014;67:451-470. doi:10.1111/bmsp.12028

36. Sobel ME. Asymptotic Confidence Intervals for Indirect Effects in Structural Equation Models. *Sociol Methodol*. 1982;13:290. doi:10.2307/270723

37. Tjur T. Coefficients of Determination in Logistic Regression Models - A New Proposal: The Coefficient of Discrimination. *Am Stat*. 2009;634(4):366-372. doi:10.1198/tast.2009.08210doi.org/10.1198/tast.2009.08210

38. Mittlböck M, Schemper M. Explained variation for logistic regression. *Stat Med*. 1996;15(19):1987-1997. doi:10.1002/(SICI)1097-0258(19961015)15:19<1987::AID-SIM318>3.0.CO;2-9

39. Hellevik O. Linear versus logistic regression when the dependent variable is a dichotomy. *Qual Quant*. 2009;43(1):59-74. doi:10.1007/s11135-007-9077-3

40. Hu LT, Bentler PM. Cutoff criteria for fit indexes in covariance structure analysis: conventional criteria versus new alternatives. *Struct Equ Model*. 1999;6:1-55.

41. Bentler PM. Comparative fit indexes in structural models. *Psychol Bull*. 1990;107:238-246.

42. Tucker LR, Lewis C. A reliability coefficient for maximum likelihood factor analysis. *Psychometrika*. 1973;38:1-10.

43. StataCorp. *Stata 13*. College Station, TX: StataCorp LP; 2013.

44. DeFranco EA, Lian M, Muglia LJ, Schootman M. Area-level poverty and preterm birth risk: A population-based multilevel analysis. *BMC Public Health*. 2008;8(1):316. doi:10.1186/1471-2458-8-316

45. Bartley M, Blane D. Inequality and social class. In: Scambler G, ed. *Sociology as Applied to Medicine*. Elsevier; 2008.

For peer review only

For peer review only - http://bmjopen.bmj.com/site/about/guidelines.xhtml

46. Newton JN, Briggs ADM, Murray CJL, et al. Changes in health in England, with analysis by English regions and areas of deprivation, 1990-2013: a systematic analysis for the Global Burden of Disease Study 2013. *Lancet*. 2015;386(10010):2257-2274. doi:10.1016/S0140-6736(15)00195-6

47. Gray R, Headley J, Oakley L, Kurinczuk JJ, Brocklehurst P, Hollowell J. *Inequalities in Infant Mortality Project Briefing Paper 3. Towards an Understanding of Variations in Infant Mortality Rates between Different Ethnic Groups in England & Wales*. Oxford; 2009. https://www.npeu.ox.ac.uk/downloads/files/infant-mortality/Infant-Mortality-Briefing-Paper-3.pdf.

48. La Placa V, Corlyon J. Barriers to inclusion and successful engagement of parents in mainstream services: evidence and research. *J Child Serv*. 2014;9(3):220-234. doi:10.1108/JCS-05-2014-0027

49. Pager D, Shepherd H. The Sociology of Discrimination: Racial Discrimination in Employment, Housing, Credit, and Consumer Markets. *Annu Rev Sociol*. 2008;34(1):181-209. doi:10.1146/annurev.soc.33.040406.131740

50. Booth AL, Leigh A, Varganova E. Does ethnic discrimination vary across minority groups? Evidence from a field experiment. *Oxf Bull Econ Stat*. 2012;74(4):547-573. doi:10.1111/j.1468-0084.2011.00664.x

51. Department for Work & Pensions. Labour Market Status by Ethnic Group: data to September 2013. 2014;(January). https://www.gov.uk/government/uploads/system/uploads/attachment_data/file/269689/labour-market-status-by-ethnic-group.pdf. Accessed March 1, 2018.

52. Schiller B. *The Economics of Poverty and Discrimination*. 9th ed. Upper Saddle River, NJ: Pearson Prentice Hall; 2004.

53. Ellis A, Fry, R. Regional health inequalities in England. *Reg Trends*. 2010;42(1):60-79. doi:10.1057/rt.2010.5

54. Marmot M, Ryff CD, Bumpass LL, Shipley M, Marks NF. Social inequalities in health: Next questions and converging evidence. *Soc Sci Med*. 1997;44(6):901-910. doi:10.1016/S0277-9536(96)00194-3

55. Cutler D, Lleras-Muney A. *Education and Health: Evaluating Theories and Evidence*. Cambridge, MA; 2006. doi:10.3386/w12352

For peer review only

For peer review only - http://bmjopen.bmj.com/site/about/guidelines.xhtml

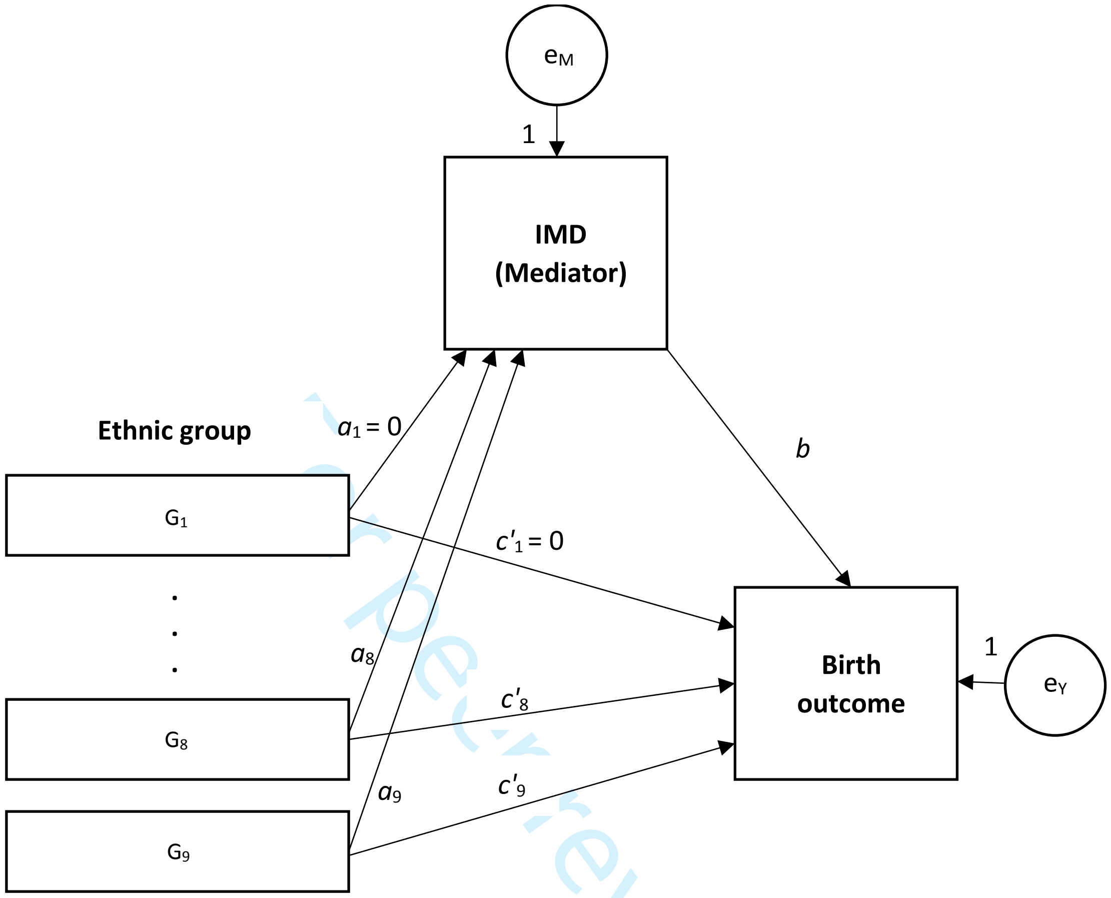

Figure 1: Mediation model for multi-categorical exposure variable

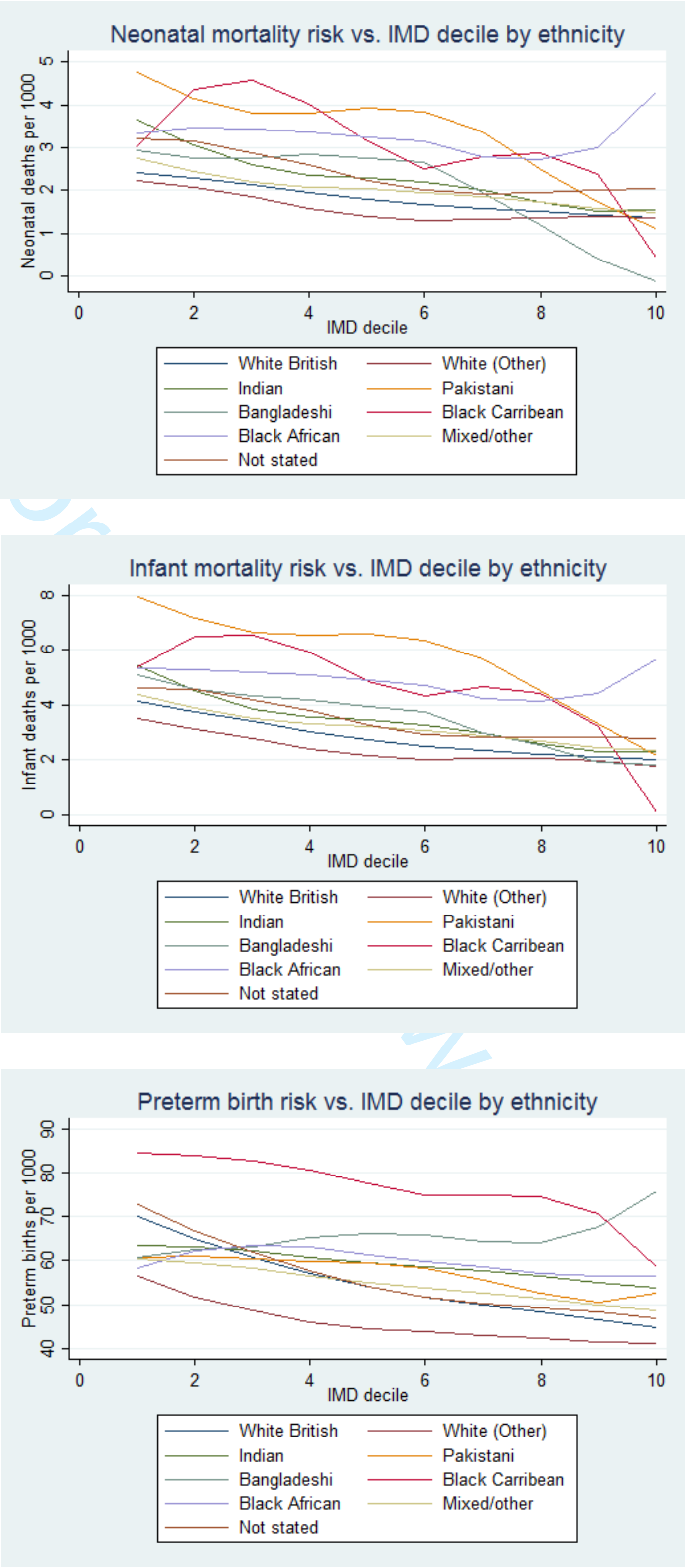

Figure 2: Distribution of neonatal mortality (top), infant mortality (centre) and preterm births (bottom) across IMD deciles within the ethnic groups, England and Wales, births 2006 – 2012

Supplementary material

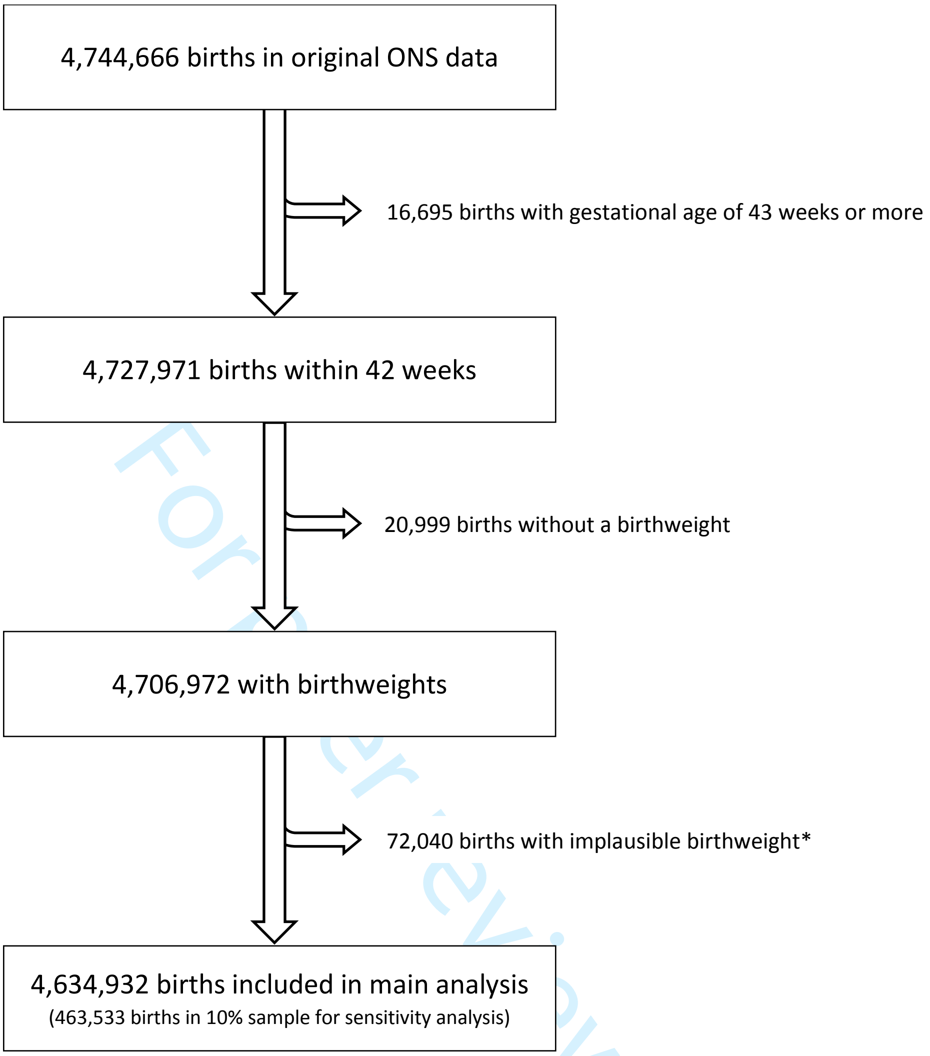

* A birthweight is considered implausible if it exceeds twice the interquartile range above and below the sex, gestation and ethnic group-specific median weight

Supplementary figure 1: Sample profile of the infants included in the analysis, England and Wales, births 2006 – 2012

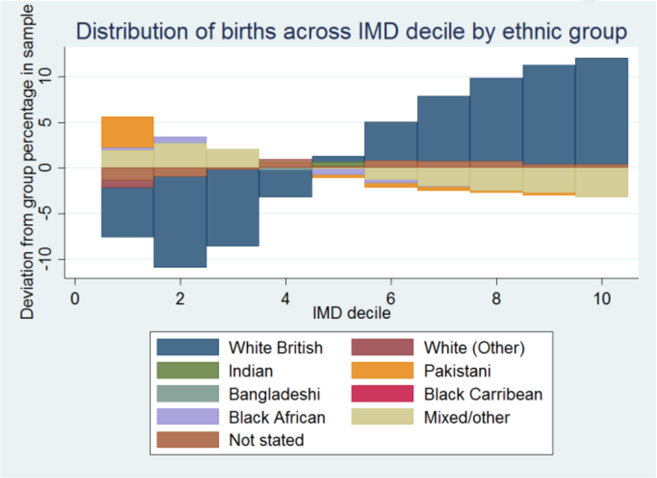

Supplementary figure 2: Distribution of births in each ethnic group and IMD relative to the ethnic group proportion of births, England and Wales, births 2006 – 2012

For peer review only - http://bmjopen.bmj.com/site/about/guidelines.xhtml

Supplementary table 1: Risks of adverse birth outcomes across ethnic groups and NS-SEC classification in the 10% sample of births, England and Wales, births 2006 – 2012

	**Risk of outcome per 1,000 live births**		
Ethnic group	**Neonatal mortality**	**Infant mortality**	**Preterm birth**
White British	1.8	2.8	55.5
White (other)	1.7	2.4	47.0
Indian	2.6	3.8	56.0
Pakistani	4.0	6.9	60.1
Bangladeshi	2.4	4.4	59.3
Black Caribbean	3.9	6.4	82.0
Black African	4.1	5.8	62.9
Mixed/other	2.3	3.7	57.4
Not stated	2.5	3.8	54.7
NS-SEC classification			
9 never worked, unemployed	3.4	5.4	74.3
8 routine occupations	2.5	4.1	64.1
7 semi-routine occupations	2.6	4.3	62.2
6 lower supervisory	2.0	3.0	57.7
5 small employers	2.0	3.5	54.6
4 intermediate occupations	2.3	3.4	56.1
3 lower managerial	1.8	2.6	51.2
2 higher professional	1.8	2.4	47.2
1 large employers	1.5	2.1	47.1
**Overall**	**2.1**	**3.3**	**55.8**

Supplementary table 2: Slope and relative indices of inequality (with bootstrap 95% confidence intervals) in the 10% sample of births, England and Wales, births 2006 – 2012

	**Neonatal mortality**	**Infant mortality**	**Preterm birth**
**Index of inequality across NS-SEC**			
Slope index	1.41<br>(0.97 – 1.85)	2.71<br>(2.14 – 3.28)	23.25<br>(20.78 – 25.71)
Relative index	1.86<br>(1.46 – 2.26)	2.19<br>(1.78 – 2.59)	1.49<br>(1.42 – 1.56)

For peer review only - http://bmjopen.bmj.com/site/about/guidelines.xhtml

STROBE Statement—Checklist of items that should be included in reports of ***cohort studies***

	Item No	Recommendation	Page No
**Title and abstract**	1	(*a*) Indicate the study's design with a commonly used term in the title or the abstract	1
		(*b*) Provide in the abstract an informative and balanced summary of what was done and what was found	2
**Introduction**			
Background/rationale	2	Explain the scientific background and rationale for the investigation being reported	4
Objectives	3	State specific objectives, including any prespecified hypotheses	4
**Methods**			
Study design	4	Present key elements of study design early in the paper	4
Setting	5	Describe the setting, locations, and relevant dates, including periods of recruitment, exposure, follow-up, and data collection	5
Participants	6	(*a*) Give the eligibility criteria, and the sources and methods of selection of participants. Describe methods of follow-up	5
		(*b*) For matched studies, give matching criteria and number of exposed and unexposed	
Variables	7	Clearly define all outcomes, exposures, predictors, potential confounders, and effect modifiers. Give diagnostic criteria, if applicable	5
Data sources/ measurement	8*	For each variable of interest, give sources of data and details of methods of assessment (measurement). Describe comparability of assessment methods if there is more than one group	5
Bias	9	Describe any efforts to address potential sources of bias	6
Study size	10	Explain how the study size was arrived at	N/A
Quantitative variables	11	Explain how quantitative variables were handled in the analyses. If applicable, describe which groupings were chosen and why	6
Statistical methods	12	(*a*) Describe all statistical methods, including those used to control for confounding	6-8
		(*b*) Describe any methods used to examine subgroups and interactions	
		(*c*) Explain how missing data were addressed	
		(*d*) If applicable, explain how loss to follow-up was addressed	
		(*e*) Describe any sensitivity analyses	
**Results**			
Participants	13*	(a) Report numbers of individuals at each stage of study—eg numbers potentially eligible, examined for eligibility, confirmed eligible, included in the study, completing follow-up, and analysed	8
		(b) Give reasons for non-participation at each stage	
		(c) Consider use of a flow diagram	
Descriptive data	14*	(a) Give characteristics of study participants (eg demographic, clinical, social) and information on exposures and potential confounders	9-10
		(b) Indicate number of participants with missing data for each variable of interest	
		(c) Summarise follow-up time (eg, average and total amount)	
Outcome data	15*	Report numbers of outcome events or summary measures over time	9-11

For peer review only

Main results	16	(*a*) Give unadjusted estimates and, if applicable, confounder-adjusted estimates and their precision (eg, 95% confidence interval). Make clear which confounders were adjusted for and why they were included<br>(*b*) Report category boundaries when continuous variables were categorized<br>(*c*) If relevant, consider translating estimates of relative risk into absolute risk for a meaningful time period	11
Other analyses	17	Report other analyses done—eg analyses of subgroups and interactions, and sensitivity analyses	11-12
**Discussion**			
Key results	18	Summarise key results with reference to study objectives	13
Limitations	19	Discuss limitations of the study, taking into account sources of potential bias or imprecision. Discuss both direction and magnitude of any potential bias	14
Interpretation	20	Give a cautious overall interpretation of results considering objectives, limitations, multiplicity of analyses, results from similar studies, and other relevant evidence	15
Generalisability	21	Discuss the generalisability (external validity) of the study results	13
**Other information**			
Funding	22	Give the source of funding and the role of the funders for the present study and, if applicable, for the original study on which the present article is based	16

*Give information separately for exposed and unexposed groups.

Note: An Explanation and Elaboration article discusses each checklist item and gives methodological background and published examples of transparent reporting. The STROBE checklist is best used in conjunction with this article (freely available on the Web sites of PLoS Medicine at http://www.plosmedicine.org/, Annals of Internal Medicine at http://www.annals.org/, and Epidemiology at http://www.epidem.com/). Information on the STROBE Initiative is available at http://www.strobe-statement.org.

For peer review only

For peer review only - http://bmjopen.bmj.com/site/about/guidelines.xhtml

BMJ Open

The joint contribution of socioeconomic circumstances and ethnic group to variations in preterm birth, neonatal mortality and infant mortality in England and Wales – a population-based retrospective cohort study using routine data from 2006 to 2012

Journal:	*BMJ Open*
Manuscript ID	bmjopen-2018-028227.R1
Article Type:	Research
Date Submitted by the Author:	16-May-2019
Complete List of Authors:	Opondo, Charles; University of Oxford, National Perinatal Epidemiology Unit<br>Gray, Ron; University of Oxford, National Perinatal Epidemiology Unit<br>Hollowell, Jennifer; University of Oxford, National Perinatal Epidemiology Unit<br>Li, Yangmei; University of Oxford, National Perinatal Epidemiology Unit<br>Kurinczuk, Jenny; University of Oxford, National Perinatal Epidemiology Unit<br>Quigley, Maria; University of Oxford, National Perinatal Epidemiology Unit
**Primary Subject Heading**:	Epidemiology
Secondary Subject Heading:	Public health
Keywords:	EPIDEMIOLOGY, PUBLIC HEALTH, PAEDIATRICS, STATISTICS & RESEARCH METHODS

SCHOLARONE™
Manuscripts

The joint contribution of socioeconomic circumstances and ethnic group to variations in preterm birth, neonatal mortality and infant mortality in England and Wales – a population-based retrospective cohort study using routine data from 2006 to 2012

Charles Opondo*, Ron Gray, Jennifer Hollowell, Yangmei Li, Jennifer J. Kurinczuk, Maria A. Quigley

Policy Research Unit in Maternal Health and Care, National Perinatal Epidemiology Unit, Nuffield Department of Population Health, University of Oxford, Oxford, United Kingdom

***Corresponding author's address**: Richard Doll Building, Roosevelt Drive, Headington, Oxford OX3 7LF. charles.opondo@npeu.ox.ac.uk

Word counts: abstract – 274; main text – 3,842; tables – 4; figures – 2.

Key words: socioeconomic circumstances, area deprivation, IMD, NS-SEC, ethnicity, neonatal mortality, infant mortality, preterm birth, mediation

For peer review only

For peer review only - http://bmjopen.bmj.com/site/about/guidelines.xhtml

Abstract

Objectives: This study aimed to describe the variation in risks of adverse birth outcomes across ethnic groups and socioeconomic circumstances, and to explore the evidence of mediation by socioeconomic circumstances of the effect of ethnicity on birth outcomes.

Setting: England and Wales.

Participants: The data came from the 4.6 million singleton live births between 2006 and 2012.

Exposure: The main exposure was ethnic group. Socioeconomic circumstances, the hypothesised mediator, was measured using the Index of Multiple Deprivation (IMD), an area-level measure of deprivation, based on the mother's place of residence.

Primary and secondary outcome measures: The primary outcomes were birth outcomes, namely: neonatal death, infant death and preterm birth. We estimated the slope and relative indices of inequality to describe differences in birth outcomes across IMD, and the proportion of the variance in birth outcomes across ethnic groups attributable to IMD. We investigated mediation by IMD on birth outcomes across ethnic groups using structural equation modelling.

Results: Neonatal mortality, infant mortality and preterm birth risks were 2.1 per 1,000, 3.2 per 1,000 and 5.6% respectively. Babies in the most deprived areas had 47% to 129% greater risk of adverse birth outcomes than those in the least deprived areas. Minority ethnic babies had 48% to 138% greater risk of adverse birth outcomes compared with white British babies. Up to a third of the variance in birth outcomes across ethnic groups was attributable to differences in IMD, and there was strong statistical evidence of an indirect effect through IMD in the effect of ethnicity on birth outcomes.

Conclusion: There is evidence that socioeconomic circumstances could be contributing to the differences in birth outcomes across ethnic groups.

For peer review only - http://bmjopen.bmj.com/site/about/guidelines.xhtml

Article summary

Strengths and limitations of the study

- This study was based on a large number of observations, which provided sufficient statistical power to investigate associations across a variety of factors and subgroups.
- The study was population-based and sufficiently large to enable individual ethnic groups to be studied.
- We used an area-level measure to quantify the socioeconomic circumstances of individuals, although we also conducted a sensitivity analysis based on an individual-level measure available in a subset of the population.
- Observational data such as these are prone to unmeasured confounding, thus despite the reporting of adjusted estimates, some potential for residual confounding by unmeasured factors remains.

For peer review only

For peer review only - http://bmjopen.bmj.com/site/about/guidelines.xhtml

Introduction

The rates of adverse birth outcomes, and indeed most other adverse health outcomes, have declined steadily over the last several decades all over the world[1–3]. These trends have been attributed to several factors including improvements in access to healthcare, nutrition, sanitation and living standards, access to birth control, literacy, and advances in medical science[1,4]. In England and Wales the three and a half decades between 1970 and 2006 saw a decline in infant mortality by over 70% from 17.7 to 5.0 deaths per 1,000 live births[5]. Trends in preterm birth rates on the other hand have been more variable: although the longer-term global trend has been one of a gradual decline, over the past two decades the rates have levelled off in some parts of Europe[6,7] and even slightly increased in others[8].

Despite these mostly positive trends, stark disparities in birth outcomes persist across some individual and group characteristics. Previous research has shown that worse socioeconomic circumstances[9], ethnic minority status[10], lone parenthood, extremes of maternal age, maternal smoking, obesity and poor access and uptake of health services[11] are all associated with poorer birth outcomes. In particular, the risk of infant mortality is higher in ethnic minority babies[12,13], as are the risks of neonatal mortality and preterm birth[10].

At the same time, past and recent patterns of migration in England and Wales and many other parts of Europe have resulted in increasing proportions of births to women from ethnic minority groups[14,15]. For example, in 2010 about 31% of all births in the Eurozone[16] and 25% in the UK[17] were to migrant women, most of whom belonged to minority ethnic groups and had higher fertility rates than the majority white groups[18]. Consequently, there has been an increase in the proportion of the population at risk of adverse birth outcomes, with a disproportionate burden borne by minority ethnic groups. This presents an increasingly significant public health issue which must be addressed[19] if we are to sustain the historical decline in most adverse birth outcomes.

Designing interventions and policies aimed at reducing health disparities requires an understanding of the mechanisms through which heterogeneity of outcomes arise[20]. Considering the role of ethnicity and relative deprivation, previous evidence highlights the role of ethnicity as a predictor of socioeconomic circumstances, with economic and social exclusion cited as examples of possible mechanisms of this association [21–23], and of socioeconomic circumstances as a predictor of adverse outcomes. This study therefore aimed to describe the variation in risks of adverse birth outcomes across ethnic groups and levels of socioeconomic circumstances, and to explore evidence for the role of socioeconomic circumstances in explaining ethnic variations in birth outcomes. We hypothesised that socioeconomic circumstances was a mediator of the effect of ethnic group on preterm birth and neonatal and infant mortality rates.

For peer review only - http://bmjopen.bmj.com/site/about/guidelines.xhtml

Methods

Study design

This was a retrospective cohort study of all singleton live births at gestational age of 22 weeks or more in England and Wales between 2006 and 2012.

Data sources, acquisition and preparation

Data from the national births and deaths registries in England and Wales have been linked since 1993. The Office for National Statistics (ONS) checks these data to minimise inconsistencies. However, these data do not include characteristics such as ethnic group and gestational age. To obtain these characteristics, the registries data have been further linked to the birth notifications made by midwives and birth attendants using the babies' National Health Service (NHS) numbers. Until 2015, birth notifications were done using a system called the NHS Numbers for Babies (NN4B)[24]. The data linkage and its evaluation have been described in detail elsewhere[24]. The ONS provided us with linked data relating to births that occurred between 1st January 2006 and 31st December 2012. Births with implausible values for gestational age, i.e. those equal to or greater than 43 weeks, and missing values for birthweight were removed from the linked dataset. Additionally, births with birthweight exceeding twice the interquartile range above or below the median birthweight of the sex-gestation-ethnic group-specific stratum were removed, as they were deemed implausible[25].

Outcomes, main explanatory variable and covariates

Outcomes of interest were: neonatal death, defined as a death from any cause occurring within the first 28 days of life; infant death, defined as a death from any cause occurring before the first birthday; and preterm birth, defined as a birth occurring before 37 completed weeks of gestation[26]. Gestational age in these data was intended to be based on last menstrual period, although it is likely that some were actually based on ultrasound estimates[27].

The main explanatory variable was ethnic group of the baby as recorded in the NN4B notification system, based on the list of ethnic categories used in the 2001 Census in England and Wales. The development of this list has been described elsewhere[28]. Nine categories were derived from those reported, namely White British, Other White, Indian, Pakistani, Bangladeshi, Black Caribbean, Black African, a 'Mixed/Other' group (which included all mixed-ethnicity groups; other Asian groups not discretely classified as Bangladeshi, Indian or Pakistani; other Black groups not discretely categorised as Black African or Black Caribbean; Chinese; and groups recorded as 'other'), and a 'Not stated' group[13]. These

For peer review only - http://bmjopen.bmj.com/site/about/guidelines.xhtml
For peer review only

categories are broadly similar to groupings that have been used in recent studies and reports[29,30]. It is uncertain as to whether the reported ethnic group was actually the baby's and not the mother's or whether it was in fact reported by the mother and not a health professional[31].

Socioeconomic circumstances was considered a potential mediator of the association between ethnicity and birth outcomes. It was assessed using the 2015 English Index of Multiple Deprivation (IMD)[32] for births in England and the 2014 Welsh IMD[33] for births in Wales. The IMD is a composite index of the relative deprivation of a defined geographical area, usually a homogeneous small area of relatively even size containing approximately 1,500 people. The index is computed from a series of indicators and statistics measuring the income, employment, health deprivation and disability, educational skills and training, housing and geographical access to services of the individuals living in an area. For the purpose of this analysis, areas were grouped into deciles of IMD, with the most deprived in the lowest decile and the least deprived in the highest decile. IMD was available for all births in the linked dataset.

An individual-level measure of socioeconomic circumstances, the National Statistics Socioeconomic Classification (NS-SEC), was available for a 10% sample of live births in which information on parental occupation recorded at birth registration was coded by the ONS. The NS-SEC is an occupationally based measure of employment relations and conditions of occupations, conceptually relevant in showing the structure of socioeconomic circumstances and explaining various social phenomena[34]. Individuals are grouped into nine categories, which are ordered starting with employment classes corresponding to better socioeconomic circumstances. To align with the direction of ordering of IMD, NS-SEC was reverse-coded so that the lowest category corresponded to the lowest socioeconomic classification and the highest category corresponded to the highest socioeconomic classification. A household NS-SEC was derived from parental occupation by assigning the higher parental NS-SEC classification to the household.

The linked dataset also included covariates such as mother's age, mother's country of birth which was grouped into UK and non-UK for this analysis, birth registration type (married parents, unmarried parents living at the same address, unmarried parents living at different addresses, or a sole registrant), year of birth between 2006 and 2012, and sex of child. These variables were included in our analyses as potential confounders of the association between socioeconomic circumstances measured using IMD and birth outcomes. Mother's age and year of birth were modelled as continuous variables and any departures from linear association with the outcomes were explored and fitted.

For peer review only - http://bmjopen.bmj.com/site/about/guidelines.xhtml

Analysis

The slope index of inequality (SII) and the relative index of inequality (RII) were estimated to describe the differences in birth outcomes across IMD[35,36]. The SII is a measure of absolute inequality which describes gradients in outcomes across subgroups with a natural ordering. It is expressed in terms of the difference between predicted outcomes of individuals in the highest versus lowest deprivation decile. The RII is a measure of relative inequality, expressed as the ratio of predicted outcome between the highest versus lowest level of deprivation. SII and RII were estimated using the method suggested in the WHO Handbook on Health Inequality Monitoring[37], which applied regression analysis – including appropriate modelling of any departures from linearity – to predict risks of birth outcomes in the most and least deprived individuals. Confidence intervals for the SII and RII were estimated by bootstrapping. As a sensitivity analysis, we repeated these analyses using household NS-SEC instead of IMD in the 10% sample of live births for which NS-SEC was available.

Two approaches were used to investigate the role of relative deprivation in the association between ethnic group and birth outcomes. First, the amount of variation in birth outcomes across ethnic groups which was explained by socioeconomic circumstances – measured using IMD deciles and modelled by linear regression – was estimated as the relative change in the residual ethnic group-level variance in outcomes as a function of relative deprivation[38,39]. The second approach, which assumed a causal link between ethnic group and IMD, and between IMD and adverse birth outcomes, involved structural equation modelling (SEM) to test an indirect effect through IMD in the effect of ethnicity on birth outcomes. This was based on the method described by Baron and Kenny[40] (Figure 1). It involved modelling the unadjusted effect of ethnic group on birth outcomes (path *c*, the *total effect,* not shown in Figure 1), and of ethnic group on IMD decile (path *a*), followed by estimation of the effect of ethnic group on birth outcomes (path *c'*) when adjusted for IMD (path *b*). Since the main exposure variable was multi-categorical, the *relative* indirect effect of IMD was estimated for each ethnic group, with white British ethnic group as the comparison group [41] followed by a Sobel test to assess the strength of evidence for an indirect effect[42]. Evidence of an indirect effect in at least one group is required to conclude overall mediation for the effect of the multi-categorical exposure[41].

The relative indirect effect, i.e. the proportion of total effect mediated, was calculated as *ab/c* or equivalently 1-(*c'/c*). A lack of evidence of effect in path *a* or *b* implied the absence of an indirect effect; a lack of evidence of effect in path *c* implied that there was no effect to be mediated. In either case, the relative indirect effect was meaningless, therefore not calculated. Linear, rather than logistic, regression was preferred for modelling all effects despite the birth outcomes being binary variables because explained variance measures in linear models are most intuitive,[43,44] as is the causal decomposition of bivariate associations in linear models, and given that the large number of observations in this linked

For peer review only - http://bmjopen.bmj.com/site/about/guidelines.xhtml

dataset, the effect estimates and standard errors from linear regression of binary outcomes are expected to be identical to those from logistic regression[45]. To correct for multiple testing, we used a stringent threshold of $p<0.001$ when assessing the evidence for direct and indirect effects. Goodness-of-fit of the SEM was tested using the root mean square error of approximation (RMSEA)[46], the comparative fit index (CFI)[47] and the Tucker-Lewis index (TLI)[48]. These indices range in value between 0 and 1. Smaller values of the RMSEA and larger values of both CFI and TLI represent better fit.

All analyses were of complete cases. Data management, manipulation and analyses were performed using Stata v13[49].

Patient and public involvement

A Patient and Public Involvement (PPI) group was formed to advise on the analysis plan, interpretation of results, and dissemination plans for this study. In particular, the group provided guidance on ethnic groupings that were used in this study.

Results

Data on 4,744,666 infants were available in the linked dataset. We sequentially excluded 16,695 infants with gestational age of 43 weeks and above, 20,999 infants with missing birthweights and 72,040 infants with implausible birthweights. A total of 4,634,932 infants with complete data in most covariates were included in the main analysis and 463,533 in the sensitivity analysis (supplementary figure 1). There were 143 infants whose mothers' country of birth could not be coded as UK or non-UK, who were dropped from the adjusted models.

Table 1 is a summary of the characteristics of the infants included in the main analysis. The infants were largely of white British ethnic origin (65%) and mostly born to UK-born mothers (76%). The proportion of births in which the child's ethnicity was not stated was 6.2% overall, although it decreased from 9.9% in 2006 to 3.3% in 2012. There were 2.6% more male than female infants in the dataset. The number of births was distributed almost evenly over the 7 years covered by the dataset, although there was a gradual year-on-year increase in the number of births. The mean age of mothers was 29 years. Over half of registrations were by married parents and almost a third were by unmarried cohabiting parents. The rest were either joint registrations by parents living separately or single-parent registrants. There were proportionately fewer infants in the less deprived IMD groups consistent with the distribution of the overall population across the deciles of IMD.

For peer review only - http://bmjopen.bmj.com/site/about/guidelines.xhtml
For peer review only

The overall proportion of preterm births was 5.6%. Infant and neonatal deaths were less common, occurring in 3.2 and 2.1 per 1,000 live births, respectively. However, more adverse birth outcomes occurred in infants born in deprived areas, overall (Table 2) and within ethnic group (Figure 2), and a disproportionate number of adverse outcomes occurred among ethnic minority infants (Table 2). Additionally, there were disproportionately more ethnic minority infants born in socio-economically deprived areas. In particular, there were higher proportions of black, Pakistani and Bangladeshi infants in the lower IMD deciles than the overall proportion of infants of these ethnicities, but a lower proportion of the same ethnicities in the higher IMD deciles; the opposite was true for white infants (Supplementary figure 2). The distribution of risks of adverse outcomes across ethnic groups in the 10% sample used for the sensitivity analysis was virtually identical to that in the overall sample (Supplementary table 1).

The slope and relative indices of inequality in IMD across birth outcomes are presented in Table 3. They show that infants born in the most socio-economically deprived areas had between 1.47 and 2.29 times the risk of adverse birth outcomes of those in the least deprived areas. The slope and relative indices of adverse birth outcomes across household NS-SEC were similar to those based on IMD: infants born in the most socio-economically deprived households had between 1.49 and 2.19 times the risk of adverse birth outcomes of those in the least deprived households (Supplementary table 2). Table 3 also shows that a small proportion, between 0.7% and 3.1%, of the overall variance in birth outcomes at ethnic group level was attributable to within-group differences. This implies that most of the variance in birth outcomes was due to between-group differences. Between 25.8% and 35.1% of the variance in outcomes at ethnic group level was explained by IMD in both crude and adjusted regression models.

There was strong evidence of an indirect effect of ethnic group on birth outcomes through socioeconomic circumstances (Sobel test p-values <0.001). Infants of all ethnic groups except white non-British infants had higher adjusted risks of neonatal and infant mortality than white British infants (Supplementary table 3). The same was true for preterm birth, except there was no evidence of a difference for Pakistani infants, and mixed/other infants had lower adjusted risk of preterm birth than white British infants. The relative indirect effects varied across ethnic groups and across outcomes, from -20.1% for infant mortality in white non-British infants to 78.2% for preterm birth in Black African infants, all relative to white British infants (Table 4). Given that the indirect effects were estimated relative to the white British group, negative indirect effect did not imply suppression as it would in absolute mediation models, but a lower indirect effect than the comparison group. The structural equation models were a good fit for the data: in all models the RMSEA was <0.001 and both CFI and TLI were >0.999.

For peer review only - http://bmjopen.bmj.com/site/about/guidelines.xhtml

Table 1: Characteristics of the 4,634,932 infants born in England and Wales, 2006 – 2012

Ethnic group, n (%)	
White British	3,009,231 (64.9)
White (other)	340,526 (7.4)
Indian	132,651 (2.9)
Pakistani	180,269 (3.9)
Bangladeshi	62,948 (1.4)
Black Caribbean	47,505 (1.0)
Black African	154,076 (3.3)
Mixed or other ethnic group	419,970 (9.1)
Ethnic group not stated	287,756 (6.2)
Sex of infant, n (%)	
Male	2,377,766 (51.3)
Female	2,257,166 (48.7)
Year of birth, n (%)	
2006	631,705 (13.6)
2007	646,902 (14.0)
2008	663,918 (14.3)
2009	659,807 (14.2)
2010	671,265 (14.5)
2011	675,075 (14.6)
2012	686,260 (14.8)
Mother's age in years, mean (SD)	29.0 (6.0)
Mother's country of birth*, n (%)	
UK	3,507,324 (75.7)
Not UK	1,127,465 (24.3)
Birth registration type, n (%)	
Registration by married parents	2,499,063 (53.9)
Joint registration by unmarried parents (same address)	1,398,935 (30.2)
Joint registration by unmarried parents (different addresses)	450,500 (9.7)
Sole registrant	286,434 (6.2)
IMD decile, n (%)	
1 (most deprived)	662,767 (14.3)
2	598,259 (12.9)
3	541,795 (11.7)
4	489,932 (10.6)
5	439,270 (9.5)
6	422,908 (9.1)
7	388,665 (8.4)
8	382,585 (8.3)
9	366,851 (7.9)
10 (least deprived)	341,900 (7.4)
Preterm birth (<37 weeks gestation), n (%)	258,515 (5.6)
Neonatal mortality, n (per 1,000)	9,638 (2.1)
Infant mortality, n (per 1,000)	15,001 (3.2)

*Excludes 143 infants whose mother's country of birth is unclear or not stated

For peer review only - http://bmjopen.bmj.com/site/about/guidelines.xhtml

Table 2: Risks of adverse birth outcomes across ethnic groups and socioeconomic circumstances, England and Wales, births 2006 – 2012

	Risk of outcome		
Ethnic group	**Neonatal mortality per 1,000**	**Infant mortality per 1,000**	**Preterm birth %**
White British	1.8	2.9	5.5
White (other)	1.6	2.5	4.6
Indian	2.4	3.6	6.0
Pakistani	4.0	6.9	6.0
Bangladeshi	2.7	4.4	6.3
Black Caribbean	4.0	6.0	8.2
Black African	3.4	5.2	6.2
Mixed/other	2.1	3.4	5.6
Not stated	2.4	3.5	5.6
Index of multiple deprivation decile			
1 – most deprived	2.8	4.7	6.7
2	2.6	4.1	6.3
3	2.5	3.8	6.0
4	2.1	3.3	5.7
5	1.9	2.9	5.3
6	1.8	2.7	5.2
7	1.6	2.4	5.0
8	1.6	2.3	4.9
9	1.5	2.2	4.8
10 – least deprived	1.5	2.1	4.5
**Overall**	**2.1**	**3.2**	**55.8**

Risks are calculated as the number of events in each group divided by the number of infants in the group multiplied by 1,000 or 100

Table 3: Slope and relative indices of inequality (bootstrap 95% confidence intervals), and amount of variation in birth outcomes across ethnic groups explained by IMD, England and Wales, births 2006 – 2012

	**Neonatal mortality**	**Infant mortality**	**Preterm birth**
**Index of inequality across IMD**			
Slope index	1.48 (1.35 – 1.60)	2.66 (2.50 – 2.82)	2.15 (2.10 – 2.21)
Relative index	2.05 (1.92 – 2.18)	2.29 (2.17 – 2.41)	1.47 (1.46 – 1.49)
**Proportion of total variance:**			
within ethnic group (crude)	2.7%	3.1%	0.7%
explained by IMD (crude)	30.1%	34.0%	28.2%
explained by IMD (adjusted*)	25.8%	27.3%	35.1%

*adjusting for mother's age, mother's country of birth, registration type, year of birth and sex of child

For peer review only - http://bmjopen.bmj.com/site/about/guidelines.xhtml

Table 4: Relative indirect effect of ethnic group on birth outcomes, with 95% confidence intervals, England and Wales, births 2006 – 2012

	Neonatal mortality	Infant mortality	Preterm birth
White British	[comparison group]	[comparison group]	[comparison group]
White (other)	_	-20.1% (-35.9 to -4.4)	-8.8% (-9.5 to -8.0)
Indian	14.7% (8.5 to 20.1)	21.0% (10.4 to 31.6)	29.7% (19.9 to 39.4)
Pakistani	14.0% (11.3 to 16.7)	13.9% (12.2 to 15.6)	_
Bangladeshi	_	40.0% (24.4 to 55.2)	_
Black Caribbean	13.4% (10.5 to 16.3)	16.8% (13.0 to 20.7)	19.3% (17.0 to 21.5)
Black African	18.6% (14.1 to 23.1)	22.6% (18.5 to 27.0)	78.2% (64.2 to 92.2)
Mixed or other	_	_	_
Not stated	1.6% (0.8 to 2.3)	2.5% (0.6 to 4.4)	_

Indirect effects are differences relative to the comparison group; they were not calculated in groups and outcomes where there was no evidence of an unadjusted effect of ethnicity on socioeconomic circumstances (path *a*) or of an adjusted effect of socioeconomic circumstances on birth outcome (path *b'*)

Discussion

These data from a cohort of babies born in England and Wales between 2006 and 2012 showed poorer birth outcomes among infants of non-white minority ethnic groups. Specifically, ethnic minority infants born over this period had up to twice the risk of adverse birth outcomes of white British infants. These data also showed that among all ethnic groups there was evidence of a gradient in birth outcomes across levels of socioeconomic circumstances, with infants born in the most deprived areas experiencing between 1.47 and 2.29 times the risk of adverse birth outcomes compared with those born in the least deprived areas. Additionally, minority ethnic infants were more likely to be born in deprived areas. Our study aimed to explore the evidence for relative deprivation as the driving mechanism for differences in birth outcomes across ethnic groups. This was based on a causal model in which we hypothesised that relative deprivation was a consequence of minority ethnic status, which was then a cause of poorer birth outcomes.

We found that between a quarter and a third of the total between-ethnic group variance in birth outcomes was explained by relative deprivation. This finding, along with the evidence of the association between ethnicity and relative deprivation, and between relative deprivation and adverse birth outcomes, is consistent with a causal link between these factors, although not sufficient proof of causality. We also found strong evidence of an indirect effect through relative deprivation in the association between ethnic group and birth outcomes, with between 13.4% and 78.2% of the risk of adverse birth outcomes in non-white minority ethnic group infants estimated to be due to the higher levels of deprivation in these groups when compared to white British infants. Compared to white non-British infants relative deprivation mediated 8.8% to 20.1% of the excess risk of adverse birth outcomes in white British infants. Together, these findings support the hypothesis that relative deprivation is one mediating mechanism through which observed differences in birth outcomes across ethnic groups arise.

For peer review only - http://bmjopen.bmj.com/site/about/guidelines.xhtml
For peer review only

There may be other mediating mechanisms involving social, demographic, cultural or even genetic factors, all of which are beyond the scope of this study, and this is further suggested by the finding of incomplete mediation across all groups and outcomes. The public health implication of these findings is that if we were to address the socioeconomic disadvantages borne by ethnic minorities in this population, we could reduce the between-ethnic group disparities in birth outcomes by between a quarter and a third in relative terms.

These conclusions are further supported by previous studies which highlight the associations between ethnicity, relative deprivation and birth outcomes. For example, other studies have shown that birth outcomes and health of infants and children tend to worsen with greater area-level poverty[50,51], lower household income[52] or in lower social classes[53,54], all of which are indicators of relative deprivation. Furthermore, previous studies in the UK[23,55] and these data also show a greater propensity for minority ethnic groups to live in socio-economically deprived areas and to suffer poorer outcomes. However, none of the studies we reviewed investigated the potential mediating effect of socioeconomic circumstances in the association between ethnicity and birth outcomes. Our study expands on previous literature by quantifying the extent to which socioeconomic circumstances contribute to ethnic disparities in birth outcomes.

We hypothesise that the relationship between ethnicity and socioeconomic circumstances, and between socioeconomic circumstances and adverse health outcomes are causal. A number of established findings support this hypothesis of causality and highlight possible mechanisms of effect. For example, language and cultural differences between minority and majority groups can create barriers to accessing or benefiting from information[56]. Minority groups are also more likely to experience various forms of discrimination based on their race, religion or culture, all of which may systematically disadvantage them in accessing services, housing and credit[57]; gaining employment[58], highlighted by the employment gap[59], and rising up the ranks while in employment[60]. In turn, poor socioeconomic circumstances can lead to poor health outcomes through: behavioural mechanisms e.g. tobacco, drug and alcohol use, poorer diet, lower utilisation of health services and contraception[9,61]; reduced access to material resources and safe environments resulting in greater mortality from pollution, poor housing and other health hazards; greater life stress caused by personal circumstances, leading to greater risk of depression, anxiety and poor mental health; with the cumulative effect of all these potential mechanisms over the life course on health and wellbeing within and across generations[62].

One limitation of this study is in the use of observational data to explore the causal relationships implied by the mediation model. Observational data are more prone to the occurrence of unmeasured confounding or reverse causality between the main exposure and the outcome, the mediator and the outcome, and between the exposure and the mediator. However, given that the exposure in this study

For peer review only - http://bmjopen.bmj.com/site/about/guidelines.xhtml

was ethnic group, there were unlikely to be any common causes or reverse causality between it and other factors. And although there was unlikely to be any reverse causality between relative deprivation and birth outcomes, this association was likely to be confounded by unmeasured factors since both factors may have had external common predictors. In our analysis, we adjusted this pathway for mother's age, mother's country of birth, birth registration type (a proxy measure for parental marital status), year of birth and sex of the child. Although this adjustment did not include factors such as education which may be common predictors of both relative deprivation and birth outcomes[63], the definition of IMD is broad and encompasses some of these factors, about which we did not have information in this dataset – a further limitation of the explanatory power of the study. We also did not have information about important parental factors such as smoking; therefore, there remains some potential for residual confounding. A criticism of the Baron and Kenny approach to the mediation analysis is that it has low statistical power[64]; however we had the benefit of a large number of observations which greatly improved our ability to detect associations. Another limitation of this study is the use of area level deprivation to model the socioeconomic circumstances of individuals; this was necessitated by the fact that individual level indicators of socioeconomic circumstances were not available for the whole sample. This has partly been addressed in the sensitivity analysis in which we have used family NS-SEC derived from parental NS-SEC as an alternative approach to measuring socioeconomic circumstances. Lastly, the ethnic group classifications used in this study may be of limited relevance to other settings.

These findings suggest that further reductions in adverse birth outcomes in England and Wales could be facilitated by interventions that reduce overall social inequality as this appears to be an important driver of variation in outcomes across ethnic groups. Such interventions must broaden their focus beyond health outcomes alone, and must aim to improve the totality of life circumstances among groups who experience the poorest outcomes.

For peer review only

For peer review only - http://bmjopen.bmj.com/site/about/guidelines.xhtml

Acknowledgements

We thank Bliss, the Race Equality Foundation, Sands, and the Lullaby Trust for their helpful involvement in the Patient and Public Involvement (PPI) consultation for this project. We also thank Alison MacFarlane, Hiranthi Jayaweera and Nirupa Dattani for their input during the planning and writing up of this study.

Author contributions

RG, JH, JJK, and MAQ conceived the study and were responsible for data acquisition. CO performed the statistical analysis with support from RG and MAQ. CO wrote the first draft, to which RG, JH, YL, JJK, and MAQ contributed and provided feedback during its development. All authors read and approved the final version of the manuscript.

Competing interests

All authors have completed the ICMJE uniform disclosure form at http://www.icmje.org/coi_disclosure.pdf and declare: no support from any organisation for the submitted work; no financial relationships with any organisations that might have an interest in the submitted work in the previous three years, no other relationships or activities that could appear to have influenced the submitted work.

Data availability

The data were provided by the Office for National Statistics (ONS) under a contractual agreement that does not permit the sharing of data. All requests for data access should be made directly to the ONS.

Ethical approval

The project was approved by the NRES Committee South Central - Oxford B, Ref 15/SC/0493.

Funding

This paper reports on an independent study which was funded by the NIHR Policy Research Programme in the Department of Health and Social Care, England (grant 108/0001). The Department of Health and

For peer review only - http://bmjopen.bmj.com/site/about/guidelines.xhtml

Social Care was not involved in any aspects of the study, and the views expressed are not necessarily those of the Department.

References

1. World Health Organization. Health across the life span. *World Heal Rep 1998*. 1998:61-111. http://www.who.int/whr/1998/en/whr98_ch3.pdf. Accessed March 8, 2018.

2. Black RE, Cousens S, Johnson HL, et al. Global, regional, and national causes of child mortality in 2008: a systematic analysis. *Lancet*. 2010;375(9730):1969-1987. doi:10.1016/S0140-6736(10)60549-1

3. Liu L, Johnson HL, Cousens S, et al. Global, regional, and national causes of child mortality: An updated systematic analysis for 2010 with time trends since 2000. *Lancet*. 2012;379:2151-2161.

4. Clift J, International Monetary Fund. *Health and Development : A Compilation of Articles from Finance & Development*. (Clift J, ed.). Washington; 2004. https://www.imf.org/external/pubs/ft/health/eng/hdwi/hdwi.pdf. Accessed March 8, 2018.

5. Norman P, Gregory I, Dorling D, Baker A. Geographical trends in infant mortality: England and Wales, 1970-2006. *Health Stat Q*. 2008;(40):18-29. https://search.proquest.com/docview/232834485/fulltextPDF/2F64288B1A974531PQ/1?accountid=13042. Accessed March 8, 2018.

6. Jakobsson M, Gissler M, Paavonen J, Tapper AM. The incidence of preterm deliveries decreases in Finland. *BJOG An Int J Obstet Gynaecol*. 2008;115(1):38-43. doi:10.1111/j.1471-0528.2007.01565.x

7. Schaaf J, Mol B, Abu-Hanna A, Ravelli A. Trends in preterm birth: singleton and multiple pregnancies in the Netherlands, 2000-2007. *BJOG An Int J Obstet Gynaecol*. 2011;118(10):1196-1204. doi:10.1111/j.1471-0528.2011.03010.x

8. Zeitlin J, Szamotulska K, Drewniak N, et al. Preterm birth time trends in Europe: A study of 19 countries. *BJOG An Int J Obstet Gynaecol*. 2013;120(11):1356-1365. doi:10.1111/1471-0528.12281

9. World Health Organisation. *Social Determinants of Health*. Second Edi. (Wilkinson R, Marmot M, eds.). Copenhagen; 2003.

10. Office for National Statistics. *Pregnancy and Ethnic Factors Influencing Births and Infant Mortality: 2013*. London; 2015. https://www.ons.gov.uk/peoplepopulationandcommunity/healthandsocialcare/causesofdeath/bulletins/pregnancyandethnicfactorsinfluencingbirthsandinfantmortality/2015-10-14.

11. The Marmot review. *The Marmot Review: Fair Society, Healthy Lives*. London; 2010. http://www.instituteofhealthequity.org/resources-reports/fair-society-healthy-lives-the-marmot-review/fair-society-healthy-lives-full-report-pdf.pdf. Accessed March 9, 2018.

12. Hollowell J, Kurinczuk JJ, Brocklehurst P, Gray R. Social and Ethnic Inequalities in Infant Mortality: A Perspective from the United Kingdom. *Semin Perinatol*. 2011;35(4):240-244. doi:10.1053/j.semperi.2011.02.021

13. Li Y, Quigley MA, Dattani N, et al. The contribution of gestational age, area deprivation and mother's country of birth to ethnic variations in infant mortality in England and Wales: A national cohort study using routinely collected data. *PLoS One*. 2018;13(4):e0195146.

For peer review only

For peer review only - http://bmjopen.bmj.com/site/about/guidelines.xhtml

doi:10.1371/journal.pone.0195146

14. Cangiano A. Net Migration as a Target for Migration Policies: A Review and Appraisal of the UK Experience. *Int Migr*. 2016;54(2):18-33. doi:10.1111/imig.12223

15. Coleman D. Immigration , Population and Ethnicity: The UK in International Perspective. *Migr Obs*. 2013:1-11. www.migrationobservatory.ox.ac.uk. Accessed March 13, 2018.

16. Zeitlin J, Mohangoo A, Delnord M. *European Perinatal Health Report: Health and Care of Pregnant Women and Babies in Europe in 2010.*; 2015. doi:10.1080/03639045.2017.1415927

17. McLaren E. Births by parents' country of birth, England and Wales: 2011. *Off Natl Stat*. 2012. https://www.ons.gov.uk/peoplepopulationandcommunity/birthsdeathsandmarriages/livebirths/bulletins/parentscountryofbirthenglandandwales/2012-08-30.

18. Coleman DA, Dubuc S. The fertility of ethnic minorities in the UK, 1960s-2006. *Popul Stud (NY)*. 2010;64(1):19-41. doi:10.1080/00324720903391201

19. Public Health England. Local action on health inequalities amongst ethnic minorities. Public Health Matters. https://publichealthmatters.blog.gov.uk/2018/08/06/local-action-on-health-inequalities-amongst-ethnic-minorities/. Published 2018. Accessed May 1, 2019.

20. Jeffries N, Zaslavsky AM, Diez Roux A V, et al. Methodological Approaches to Understanding Causes of Health Disparities. *Am J Public Health*. 2019;109(S1):S28-S33. doi:10.2105/AJPH.2018.304843

21. Nazroo JY. The structuring of ethnic inequalities in health: Economic position, racial discrimination, and racism. *Am J Public Health*. 2003;93(2):277-284. doi:10.2105/AJPH.93.2.277

22. Karlsen S, Nazroo JY. Relation between racial discrimination, social class, and health among ethnic minority groups. *Am J Public Health*. 2002;92(4):624-631. doi:10.2105/AJPH.92.4.624

23. Nazroo J, Karlsen S. Ethnic inequalities in health: social class, racism and identity. *Res Find*. 2001;10(10). http://www.lancaster.ac.uk/fass/projects/hvp/pdf/fd10.pdf. Accessed December 9, 2017.

24. Hilder L, Moser K, Dattani N, Macfarlane A. Pilot linkage of NHS Numbers for Babies data with birth registrations. *Heal Stat Q*. 2007;(33):25-33. https://pdfs.semanticscholar.org/128c/a2310d5d5028d5a5cc9a69d27dc3ac20f385.pdf. Accessed March 13, 2018.

25. Tukey J. *Exploratory Data Analysis*. Addison-Wesley; 1977. https://pdfs.semanticscholar.org/2f40/fd06bc9fd00a27437e14ed171e96e4fd9326.pdf. Accessed February 1, 2018.

26. World Health Organization. *International Statistical Classification of Diseases and Related Health Problems - 10th Revision (ICD10)*. WHO; 2010. http://apps.who.int/classifications/icd10/browse/Content/statichtml/ICD10Volume2_en_2010.pdf.

27. Moser K, Hilder L. Assessing quality of NHS Numbers for Babies data and providing gestational age statistics. *Health Stat Q*. 2008;37:15-23. http://researchonline.lshtm.ac.uk/24366/.

28. Office for National Statistics. *Ethnic Group Statistics: A Guide for the Collection and Classification of Ethnicity Data*. London; 2003. http://scholar.google.com/scholar?hl=en&btnG=Search&q=intitle:Ethnic+group+statistics+A+guide+for+the+collection+and+classification+of+ethnicity+data#1.

29. Kurinczuk JJ, Hollowell J, Brocklehurst P, Gray R. *Inequalities in Infant Mortality Project Briefing*

For peer review only - http://bmjopen.bmj.com/site/about/guidelines.xhtml
For peer review only

Paper 1. Infant Mortality: Overview and Context. Oxford; 2009. https://www.npeu.ox.ac.uk/downloads/files/infant-mortality/Infant-Mortality-Briefing-Paper-1.pdf.

30. Bosveld K, Connolly H, Rendall M. *A Guide to Comparing 1991 and 2001 Census Ethnic Group Data.* London; 2006. http://webarchive.nationalarchives.gov.uk/20151014015820/http://www.ons.gov.uk/ons/rel/ethnicity/focus-on-ethnicity-and-identity/a-guide-to-comparing-1991-and-2001-census-ethnic-group-data/comparing-1991-and-2001-census-ethnic-group-data.pdf.

31. Moser K, Stanfield K, Leon D. Birthweight and gestational age by ethnic group, England and Wales 2005: introducing new data on births. *Health Stat Q*. 2008;(39):22-31, 34-55. http://www.ncbi.nlm.nih.gov/pubmed/18810886. Accessed November 27, 2017.

32. Smith T, Noble M, Noble S, Wright G, McLennan D, Plunkett E. *The English Indices of Deprivation 2015 - Technical Report*.; 2015. doi:http://dx.doi.org/http://www.communities.gov.uk/publications/corporate/statistics/indices2010technicalreport

33. Welsh Government. Welsh Index of Multiple Deprivation (WIMD) 2014. *Off Natl Stat*. 2014:104. doi:10.1136/vr.h476

34. Office for National Statistics. The National Statistics Socio-Economic Classification. https://www.ons.gov.uk/methodology/classificationsandstandards/otherclassifications/thenationalstatisticssocioeconomicclassificationnssecrebasedonsoc2010. Published 2016. Accessed June 14, 2018.

35. Regidor E. Measures of health inequalities: part 2. *J Epidemiol Community Heal*. 2004;58(11):900-903. doi:10.1136/jech.2004.023036

36. Pamuk ER. Social-class inequality in infant mortality in England and Wales from 1921 to 1980. *Eur J Popul*. 1988;4(1):1-21. doi:10.1007/BF01797104

37. WHO. Handbook on health inequality monitoring: with a special focus on low- and middle-income countries. http://apps.who.int/iris/bitstream/10665/85345/1/9789241548632_eng.pdf. Accessed March 13, 2017.

38. LaHuis DM, Hartman MJ, Hakoyama S, Clark PC. Explained variance measures for multilevel models. *Organ Res Methods*. 2014;17(4):433-451. doi:10.1177/1094428114541701

39. Snijders T, Bosker R. Modeled variance in two-level models. *Sociol Methods Res*. 1994;22(3):342-363. doi:10.1177/0049124194022003004

40. Baron RM, Kenny DA. The moderator-mediator variable distinction in social psychological research: conceptual, strategic, and statistical considerations. *J Pers Soc Psychol*. 1986;51(6):1173-1182. doi:10.1037/0022-3514.51.6.1173

41. Hayes AF, Preacher KJ. Statistical mediation analysis with a multicategorical independent variable. *Br J Math Stat Psychol*. 2014;67:451-470. doi:10.1111/bmsp.12028

42. Sobel ME. Asymptotic Confidence Intervals for Indirect Effects in Structural Equation Models. *Sociol Methodol*. 1982;13:290. doi:10.2307/270723

43. Tjur T. Coefficients of Determination in Logistic Regression Models - A New Proposal: The Coefficient of Discrimination. *Am Stat*. 2009;634(4):366-372. doi:10.1198/tast.2009.08210doi.org/10.1198/tast.2009.08210

44. Mittlböck M, Schemper M. Explained variation for logistic regression. *Stat Med*.

For peer review only - http://bmjopen.bmj.com/site/about/guidelines.xhtml

1996;15(19):1987-1997. doi:10.1002/(SICI)1097-0258(19961015)15:19<1987::AID-SIM318>3.0.CO;2-9

45. Hellevik O. Linear versus logistic regression when the dependent variable is a dichotomy. *Qual Quant*. 2009;43(1):59-74. doi:10.1007/s11135-007-9077-3

46. Hu LT, Bentler PM. Cutoff criteria for fit indexes in covariance structure analysis: conventional criteria versus new alternatives. *Struct Equ Model*. 1999;6:1-55.

47. Bentler PM. Comparative fit indexes in structural models. *Psychol Bull*. 1990;107:238-246.

48. Tucker LR, Lewis C. A reliability coefficient for maximum likelihood factor analysis. *Psychometrika*. 1973;38:1-10.

49. StataCorp. *Stata 13*. College Station, TX: StataCorp LP; 2013.

50. DeFranco EA, Lian M, Muglia LJ, Schootman M. Area-level poverty and preterm birth risk: A population-based multilevel analysis. *BMC Public Health*. 2008;8(1):316. doi:10.1186/1471-2458-8-316

51. Bonet M, Smith LK, Pilkington H, Draper ES, Zeitlin J. Neighbourhood deprivation and very preterm birth in an English and French cohort. *BMC Pregnancy Childbirth*. 2013;13(1):97. doi:10.1186/1471-2393-13-97

52. Opatowski M, Blondel B, Khoshnood B, Saurel-Cubizolles MJ. New index of social deprivation during pregnancy: Results from a national study in France. *BMJ Open*. 2016;6(4):e009511. doi:10.1136/bmjopen-2015-009511

53. Bartley M, Blane D. Inequality and social class. In: Scambler G, ed. *Sociology as Applied to Medicine*. Elsevier; 2008.

54. Poeran J, Maas AFG, Birnie E, Denktas S, Steegers EAP, Bonsel GJ. Social deprivation and adverse perinatal outcomes among Western and non-Western pregnant women in a Dutch urban population. *Soc Sci Med*. 2013;83:42-49. doi:10.1016/j.socscimed.2013.02.008

55. Gray R, Headley J, Oakley L, Kurinczuk JJ, Brocklehurst P, Hollowell J. *Inequalities in Infant Mortality Project Briefing Paper 3. Towards an Understanding of Variations in Infant Mortality Rates between Different Ethnic Groups in England & Wales*. Oxford; 2009. https://www.npeu.ox.ac.uk/downloads/files/infant-mortality/Infant-Mortality-Briefing-Paper-3.pdf.

56. La Placa V, Corlyon J. Barriers to inclusion and successful engagement of parents in mainstream services: evidence and research. *J Child Serv*. 2014;9(3):220-234. doi:10.1108/JCS-05-2014-0027

57. Pager D, Shepherd H. The Sociology of Discrimination: Racial Discrimination in Employment, Housing, Credit, and Consumer Markets. *Annu Rev Sociol*. 2008;34(1):181-209. doi:10.1146/annurev.soc.33.040406.131740

58. Booth AL, Leigh A, Varganova E. Does ethnic discrimination vary across minority groups? Evidence from a field experiment. *Oxf Bull Econ Stat*. 2012;74(4):547-573. doi:10.1111/j.1468-0084.2011.00664.x

59. Department for Work & Pensions. Labour Market Status by Ethnic Group: data to September 2013. 2014;(January). https://www.gov.uk/government/uploads/system/uploads/attachment_data/file/269689/labour-market-status-by-ethnic-group.pdf. Accessed March 1, 2018.

60. Schiller B. *The Economics of Poverty and Discrimination*. 9th ed. Upper Saddle River, NJ: Pearson Prentice Hall; 2004.

For peer review only

For peer review only - http://bmjopen.bmj.com/site/about/guidelines.xhtml

61. Ellis A, Fry, R. Regional health inequalities in England. *Reg Trends*. 2010;42(1):60-79. doi:10.1057/rt.2010.5

62. Marmot M, Ryff CD, Bumpass LL, Shipley M, Marks NF. Social inequalities in health: Next questions and converging evidence. *Soc Sci Med*. 1997;44(6):901-910. doi:10.1016/S0277-9536(96)00194-3

63. Cutler D, Lleras-Muney A. *Education and Health: Evaluating Theories and Evidence*. Cambridge, MA; 2006. doi:10.3386/w12352

64. Hayes AF. Beyond Baron and Kenny: Statistical Mediation Analysis in the New Millennium. *Commun Monogr*. 2009;76(4):408-420. doi:10.1080/03637750903310360

For peer review only

For peer review only - http://bmjopen.bmj.com/site/about/guidelines.xhtml

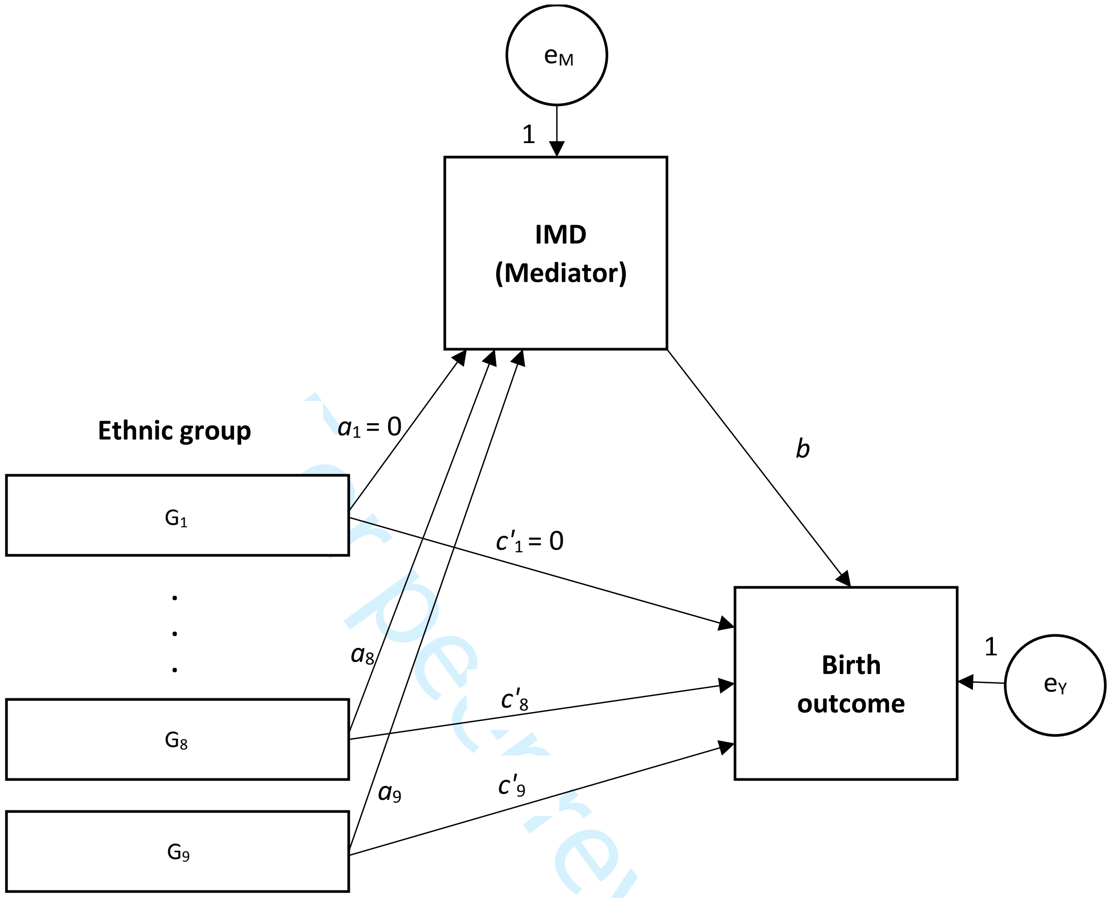

Figure 1: Mediation model for multi-categorical exposure variable

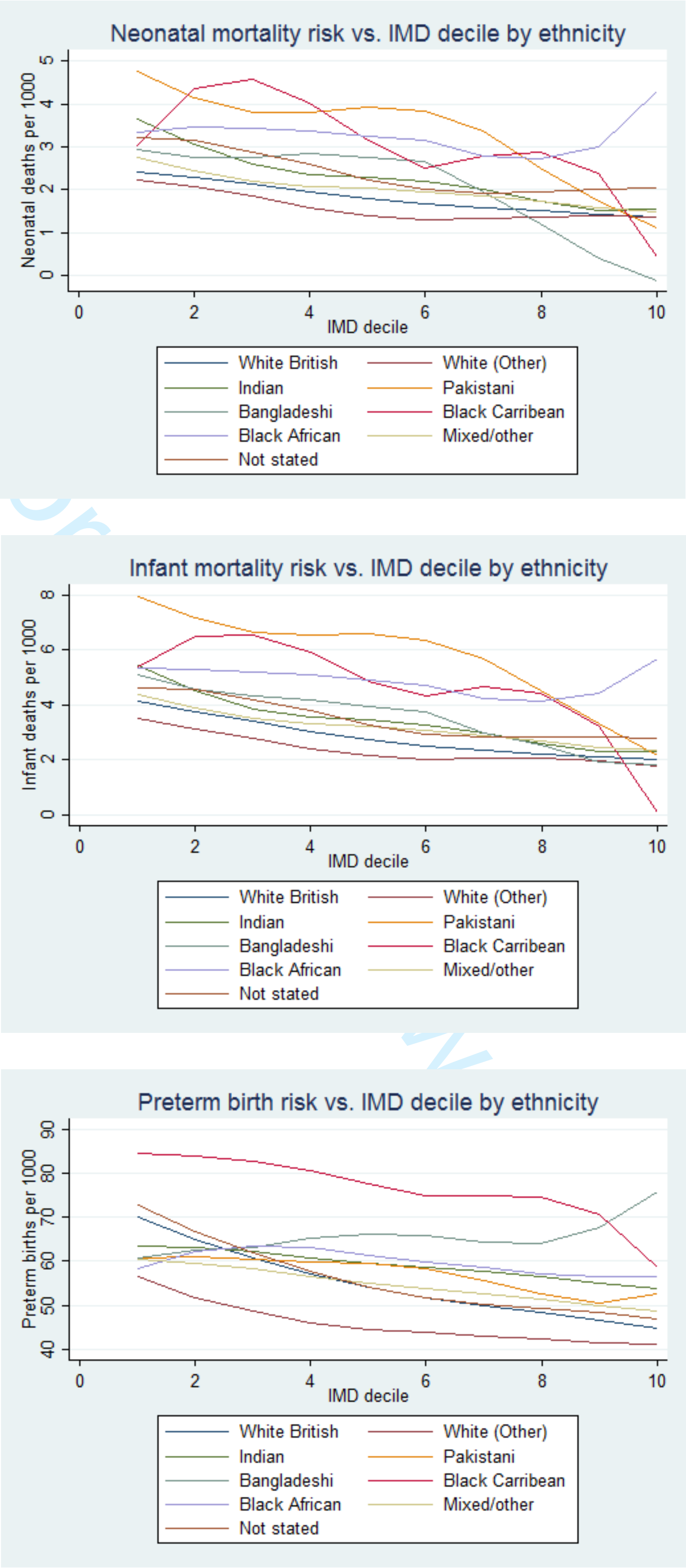

Figure 2: Distribution of neonatal mortality (top), infant mortality (centre) and preterm births (bottom) across IMD deciles within the ethnic groups, England and Wales, births 2006 – 2012

Supplementary material

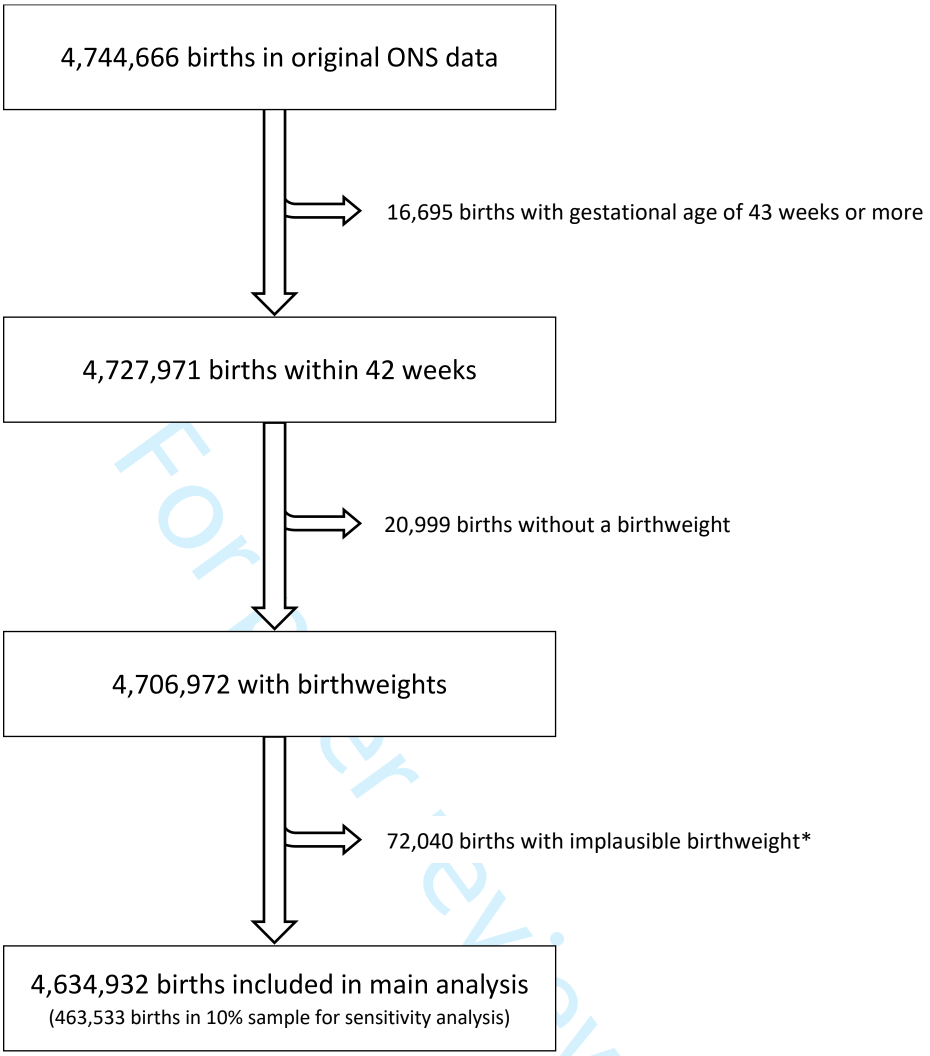

* A birthweight is considered implausible if it exceeds twice the interquartile range above and below the sex, gestation and ethnic group-specific median weight

Supplementary figure 1: Sample profile of the infants included in the analysis, England and Wales, births 2006 – 2012

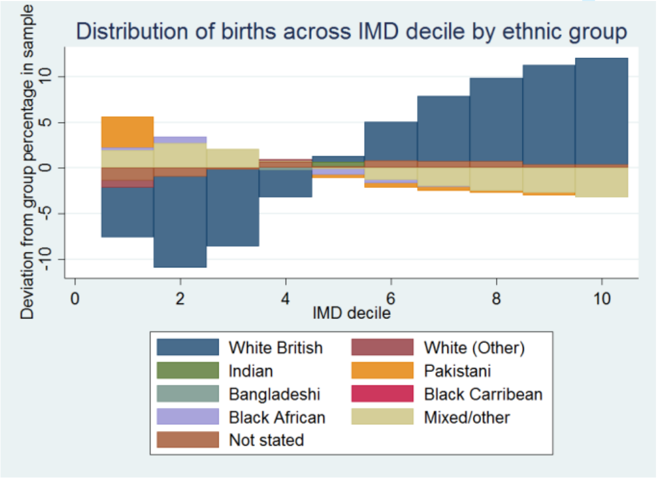

Supplementary figure 2: Distribution of births in each ethnic group and IMD relative to the ethnic group proportion of births, England and Wales, births 2006 – 2012

For peer review only - http://bmjopen.bmj.com/site/about/guidelines.xhtml

Supplementary table 1: Risks of adverse birth outcomes across ethnic groups and NS-SEC classification in the 10% sample of births, England and Wales, births 2006 – 2012

	**Risk of outcome**		
Ethnic group	**Neonatal mortality per 1,000**	**Infant mortality per 1,000**	**Preterm birth %**
White British	1.8	2.8	5.6
White (other)	1.7	2.4	4.7
Indian	2.6	3.8	5.6
Pakistani	4.0	6.9	6.0
Bangladeshi	2.4	4.4	5.9
Black Caribbean	3.9	6.4	8.2
Black African	4.1	5.8	6.3
Mixed/other	2.3	3.7	5.7
Not stated	2.5	3.8	5.5
NS-SEC classification			
9 never worked, unemployed	3.4	5.4	7.4
8 routine occupations	2.5	4.1	6.4
7 semi-routine occupations	2.6	4.3	6.2
6 lower supervisory	2.0	3.0	5.8
5 small employers	2.0	3.5	5.5
4 intermediate occupations	2.3	3.4	5.6
3 lower managerial	1.8	2.6	5.1
2 higher professional	1.8	2.4	4.7
1 large employers	1.5	2.1	4.7
**Overall**	**2.1**	**3.3**	**5.6**

Supplementary table 2: Slope and relative indices of inequality (with bootstrap 95% confidence intervals) in the 10% sample of births, England and Wales, births 2006 – 2012

**Index of inequality across NS-SEC**	**Neonatal mortality per 1,000**	**Infant mortality per 1,000**	**Preterm birth %**
Slope index	1.41 (0.97 – 1.85)	2.71 (2.14 – 3.28)	2.33 (2.08 – 2.57)
Relative index	1.86 (1.46 – 2.26)	2.19 (1.78 – 2.59)	1.49 (1.42 – 1.56)

For peer review only - http://bmjopen.bmj.com/site/about/guidelines.xhtml

Supplementary table 3: Differences in risks of adverse birth outcomes across ethnic groups, England and Wales, births 2006 – 2012

**Ethnic group**	**Difference in risk of outcome (95% CI)**					
	**Neonatal mortality per 1,000**		**Infant mortality per 1,000**		**Preterm birth %**	
	**Unadjusted**	**Adjusted**	**Unadjusted**	**Adjusted**	**Unadjusted**	**Adjusted**
White British	[comparison group]	[comparison group]	[comparison group]	[comparison group]	[comparison group]	[comparison group]
White (other)	-0.2 (-0.3 – -0.1)	-0.3 (-0.4 – -0.1)	-0.4 (-0.6 – -0.2)	-0.5 (-0.7 – -0.3)	-0.9 (-1.0 – -0.8)	-1.0 (-1.1 – -0.9)
Indian	0.5 (0.3 – 0.8)	0.4 (0.2 – 0.7)	0.7 (0.4 – 1.0)	0.6 (0.2 – 0.9)	0.5 (0.4 – 0.6)	0.3 (0.2 – 0.5)
Pakistani	2.2 (2.0 – 2.4)	1.9 (1.7 – 2.1)	4.0 (3.8 – 4.3)	3.5 (3.2 – 3.8)	0.5 (0.4 – 0.6)	-0.1 (-0.2 – 0.0)
Bangladeshi	0.9 (0.5 – 1.2)	0.5 (0.2 – 0.9)	1.5 (1.1 – 2.0)	0.9 (0.5 – 1.4)	0.8 (0.6 – 0.9)	0.2 (-0.0 – 0.3)
Black Caribbean	2.2 (1.7 –2.6)	1.9 (1.5 – 2.3)	3.1 (2.6 – 3.6)	2.6 (2.1 – 3.1)	2.7 (2.5 – 2.9)	2.2 (1.9 – 2.4)
Black African	1.5 (1.3 – 1.8)	1.2 (1.0 – 1.5)	2.3 (2.0 – 2.6)	1.8 (1.5 – 2.1)	0.6 (0.5 – 0.8)	0.1 (0.0 – 0.3)
Mixed/other	0.3 (0.2 – 0.4)	0.2 (0.0 – 0.3)	0.5 (0.4 – 0.7)	0.3 (0.1 – 0.5)	0.1 (0.0 – 0.2)	-0.1 (-0.2 – -0.1)
Not stated	0.6 (0.4 – 0.8)	0.6 (0.4 – 0.8)	0.7 (0.5 – 0.9)	0.6 (0.4 – 0.9)	0.1 (0.0 – 0.2)	0.1 (-0.0 – 0.2)

Unadjusted difference in risk of outcomes represent the total effect (path c) of ethnic group on birth outcomes; differences adjusted for socioeconomic circumstances represent the direct effect (path c')

For peer review only

For peer review only - http://bmjopen.bmj.com/site/about/guidelines.xhtml

STROBE Statement—Checklist of items that should be included in reports of ***cohort studies***

	Item No	Recommendation	Page No
**Title and abstract**	1	(*a*) Indicate the study's design with a commonly used term in the title or the abstract	1
		(*b*) Provide in the abstract an informative and balanced summary of what was done and what was found	2
**Introduction**			
Background/rationale	2	Explain the scientific background and rationale for the investigation being reported	4
Objectives	3	State specific objectives, including any prespecified hypotheses	4
**Methods**			
Study design	4	Present key elements of study design early in the paper	5
Setting	5	Describe the setting, locations, and relevant dates, including periods of recruitment, exposure, follow-up, and data collection	5
Participants	6	(*a*) Give the eligibility criteria, and the sources and methods of selection of participants. Describe methods of follow-up	5
		(*b*) For matched studies, give matching criteria and number of exposed and unexposed	
Variables	7	Clearly define all outcomes, exposures, predictors, potential confounders, and effect modifiers. Give diagnostic criteria, if applicable	5-6
Data sources/ measurement	8*	For each variable of interest, give sources of data and details of methods of assessment (measurement). Describe comparability of assessment methods if there is more than one group	5-6
Bias	9	Describe any efforts to address potential sources of bias	6
Study size	10	Explain how the study size was arrived at	N/A
Quantitative variables	11	Explain how quantitative variables were handled in the analyses. If applicable, describe which groupings were chosen and why	6
Statistical methods	12	(*a*) Describe all statistical methods, including those used to control for confounding	7-8
		(*b*) Describe any methods used to examine subgroups and interactions	
		(*c*) Explain how missing data were addressed	
		(*d*) If applicable, explain how loss to follow-up was addressed	
		(*e*) Describe any sensitivity analyses	
**Results**			
Participants	13*	(a) Report numbers of individuals at each stage of study—eg numbers potentially eligible, examined for eligibility, confirmed eligible, included in the study, completing follow-up, and analysed	8
		(b) Give reasons for non-participation at each stage	
		(c) Consider use of a flow diagram	
Descriptive data	14*	(a) Give characteristics of study participants (eg demographic, clinical, social) and information on exposures and potential confounders	9-10
		(b) Indicate number of participants with missing data for each variable of interest	
		(c) Summarise follow-up time (eg, average and total amount)	
Outcome data	15*	Report numbers of outcome events or summary measures over time	9-11

For peer review only

Main results	16	(*a*) Give unadjusted estimates and, if applicable, confounder-adjusted estimates and their precision (eg, 95% confidence interval). Make clear which confounders were adjusted for and why they were included<br>(*b*) Report category boundaries when continuous variables were categorized<br>(*c*) If relevant, consider translating estimates of relative risk into absolute risk for a meaningful time period	9-11
Other analyses	17	Report other analyses done—eg analyses of subgroups and interactions, and sensitivity analyses	9-11
**Discussion**			
Key results	18	Summarise key results with reference to study objectives	12
Limitations	19	Discuss limitations of the study, taking into account sources of potential bias or imprecision. Discuss both direction and magnitude of any potential bias	13-14
Interpretation	20	Give a cautious overall interpretation of results considering objectives, limitations, multiplicity of analyses, results from similar studies, and other relevant evidence	14
Generalisability	21	Discuss the generalisability (external validity) of the study results	14
**Other information**			
Funding	22	Give the source of funding and the role of the funders for the present study and, if applicable, for the original study on which the present article is based	15

*Give information separately for exposed and unexposed groups.

Note: An Explanation and Elaboration article discusses each checklist item and gives methodological background and published examples of transparent reporting. The STROBE checklist is best used in conjunction with this article (freely available on the Web sites of PLoS Medicine at http://www.plosmedicine.org/, Annals of Internal Medicine at http://www.annals.org/, and Epidemiology at http://www.epidem.com/). Information on the STROBE Initiative is available at http://www.strobe-statement.org.

For peer review only

BMJ Open

The joint contribution of socioeconomic circumstances and ethnic group to variations in preterm birth, neonatal mortality and infant mortality in England and Wales – a population-based retrospective cohort study using routine data from 2006 to 2012

Journal:	*BMJ Open*
Manuscript ID	bmjopen-2018-028227.R2
Article Type:	Research
Date Submitted by the Author:	14-Jul-2019
Complete List of Authors:	Opondo, Charles; University of Oxford, National Perinatal Epidemiology Unit<br>Gray, Ron; University of Oxford, National Perinatal Epidemiology Unit<br>Hollowell, Jennifer; University of Oxford, National Perinatal Epidemiology Unit<br>Li, Yangmei; University of Oxford, National Perinatal Epidemiology Unit<br>Kurinczuk, Jenny; University of Oxford, National Perinatal Epidemiology Unit<br>Quigley, Maria; University of Oxford, National Perinatal Epidemiology Unit
**Primary Subject Heading**:	Epidemiology
Secondary Subject Heading:	Public health
Keywords:	EPIDEMIOLOGY, PUBLIC HEALTH, PAEDIATRICS, STATISTICS & RESEARCH METHODS

SCHOLARONE™
Manuscripts

The joint contribution of socioeconomic circumstances and ethnic group to variations in preterm birth, neonatal mortality and infant mortality in England and Wales – a population-based retrospective cohort study using routine data from 2006 to 2012

Charles Opondo*, Ron Gray, Jennifer Hollowell, Yangmei Li, Jennifer J. Kurinczuk, Maria A. Quigley

Policy Research Unit in Maternal Health and Care, National Perinatal Epidemiology Unit, Nuffield Department of Population Health, University of Oxford, Oxford, United Kingdom

***Corresponding author's address**: Richard Doll Building, Roosevelt Drive, Headington, Oxford OX3 7LF. charles.opondo@npeu.ox.ac.uk

Word counts: abstract – 274; main text – 3,896; tables – 4; figures – 2.

Key words: socioeconomic circumstances, area deprivation, IMD, NS-SEC, ethnicity, neonatal mortality, infant mortality, preterm birth, mediation

For peer review only

For peer review only - http://bmjopen.bmj.com/site/about/guidelines.xhtml

Abstract

Objectives: This study aimed to describe the variation in risks of adverse birth outcomes across ethnic groups and socioeconomic circumstances, and to explore the evidence of mediation by socioeconomic circumstances of the effect of ethnicity on birth outcomes.

Setting: England and Wales.

Participants: The data came from the 4.6 million singleton live births between 2006 and 2012.

Exposure: The main exposure was ethnic group. Socioeconomic circumstances, the hypothesised mediator, was measured using the Index of Multiple Deprivation (IMD), an area-level measure of deprivation, based on the mother's place of residence.

Primary and secondary outcome measures: The primary outcomes were birth outcomes, namely: neonatal death, infant death and preterm birth. We estimated the slope and relative indices of inequality to describe differences in birth outcomes across IMD, and the proportion of the variance in birth outcomes across ethnic groups attributable to IMD. We investigated mediation by IMD on birth outcomes across ethnic groups using structural equation modelling.

Results: Neonatal mortality, infant mortality and preterm birth risks were 2.1 per 1,000, 3.2 per 1,000 and 5.6% respectively. Babies in the most deprived areas had 47% to 129% greater risk of adverse birth outcomes than those in the least deprived areas. Minority ethnic babies had 48% to 138% greater risk of adverse birth outcomes compared with white British babies. Up to a third of the variance in birth outcomes across ethnic groups was attributable to differences in IMD, and there was strong statistical evidence of an indirect effect through IMD in the effect of ethnicity on birth outcomes.

Conclusion: There is evidence that socioeconomic circumstances could be contributing to the differences in birth outcomes across ethnic groups.

For peer review only

For peer review only - http://bmjopen.bmj.com/site/about/guidelines.xhtml

Article summary

Strengths and limitations of the study

- This study was based on a large number of observations, which provided sufficient statistical power to investigate associations across a variety of factors and subgroups.
- The study was population-based and sufficiently large to enable individual ethnic groups to be studied.
- We used an area-level measure to quantify the socioeconomic circumstances of individuals, although we also conducted a sensitivity analysis based on an individual-level measure available in a subset of the population.
- Observational data such as these are prone to unmeasured confounding, thus despite the reporting of adjusted estimates, some potential for residual confounding by unmeasured factors remains.

For peer review only

For peer review only - http://bmjopen.bmj.com/site/about/guidelines.xhtml

Introduction

The rates of adverse birth outcomes, and indeed most other adverse health outcomes, have declined steadily over the last several decades all over the world[1–3]. These trends have been attributed to several factors including improvements in access to healthcare, nutrition, sanitation and living standards, access to birth control, literacy, and advances in medical science[1,4]. In England and Wales the three and a half decades between 1970 and 2006 saw a decline in infant mortality by over 70% from 17.7 to 5.0 deaths per 1,000 live births[5]. Trends in preterm birth rates on the other hand have been more variable: although the longer-term global trend has been one of a gradual decline, over the past two decades the rates have levelled off in some parts of Europe[6,7] and even slightly increased in others[8].

Despite these mostly positive trends, stark disparities in birth outcomes persist across some individual and group characteristics. Previous research has shown that worse socioeconomic circumstances[9], ethnic minority status[10], lone parenthood, extremes of maternal age, maternal smoking, obesity and poor access and uptake of health services[11] are all associated with poorer birth outcomes. In particular, the risk of infant mortality is higher in ethnic minority babies[12,13], as are the risks of neonatal mortality and preterm birth[10].

At the same time, past and recent patterns of migration in England and Wales and many other parts of Europe have resulted in increasing proportions of births to women from ethnic minority groups[14,15]. For example, in 2010 about 31% of all births in the Eurozone[16] and 25% in the UK[17] were to migrant women, most of whom belonged to minority ethnic groups and had higher fertility rates than the majority white groups[18]. Consequently, there has been an increase in the proportion of the population at risk of adverse birth outcomes, with a disproportionate burden borne by minority ethnic groups. This presents an increasingly significant public health issue which must be addressed[19] if we are to sustain the historical decline in most adverse birth outcomes.

Designing interventions and policies aimed at reducing health disparities requires an understanding of the mechanisms through which heterogeneity of outcomes arise[20]. Considering the role of ethnicity and relative deprivation, previous evidence highlights the role of ethnicity as a predictor of socioeconomic circumstances, with economic and social exclusion cited as examples of possible mechanisms of this association [21–23], and of socioeconomic circumstances as a predictor of adverse outcomes. This study therefore aimed to describe the variation in risks of adverse birth outcomes across ethnic groups and levels of socioeconomic circumstances, and to explore evidence for the role of socioeconomic circumstances in explaining ethnic variations in birth outcomes. We hypothesised that socioeconomic circumstances was a mediator of the effect of ethnic group on preterm birth and neonatal and infant mortality rates.

For peer review only - http://bmjopen.bmj.com/site/about/guidelines.xhtml

Methods

Study design

This was a retrospective cohort study of all singleton live births at gestational age of 22 weeks or more in England and Wales between 2006 and 2012.

Data sources, acquisition and preparation

Data from the national births and deaths registries in England and Wales have been linked since 1993. The Office for National Statistics (ONS) checks these data to minimise inconsistencies. However, these data do not include characteristics such as ethnic group and gestational age. To obtain these characteristics, the registries data have been further linked to the birth notifications made by midwives and birth attendants using the babies' National Health Service (NHS) numbers. Until 2015, birth notifications were done using a system called the NHS Numbers for Babies (NN4B)[24]. The data linkage and its evaluation have been described in detail elsewhere[24]. The ONS provided us with linked data relating to births that occurred between 1st January 2006 and 31st December 2012. Births with implausible values for gestational age, i.e. those equal to or greater than 43 weeks, and missing values for birthweight were removed from the linked dataset. Additionally, births with birthweight exceeding twice the interquartile range above or below the median birthweight of the sex-gestation-ethnic group-specific stratum were removed, as they were deemed implausible[25].

Outcomes, main explanatory variable and covariates

Outcomes of interest were: neonatal death, defined as a death from any cause occurring within the first 28 days of life; infant death, defined as a death from any cause occurring before the first birthday; and preterm birth, defined as a birth occurring before 37 completed weeks of gestation[26]. Gestational age in these data was intended to be based on last menstrual period, although it is likely that some were actually based on ultrasound estimates[27].

The main explanatory variable was ethnic group of the baby as recorded in the NN4B notification system, based on the list of ethnic categories used in the 2001 Census in England and Wales. The development of this list has been described elsewhere[28]. Nine categories were derived from those reported, namely White British, Other White, Indian, Pakistani, Bangladeshi, Black Caribbean, Black African, a 'Mixed/Other' group (which included all mixed-ethnicity groups; other Asian groups not discretely classified as Bangladeshi, Indian or Pakistani; other Black groups not discretely categorised as Black African or Black Caribbean; Chinese; and groups recorded as 'other'), and a 'Not stated' group[13]. These

For peer review only - http://bmjopen.bmj.com/site/about/guidelines.xhtml

categories are broadly similar to groupings that have been used in recent studies and reports[29,30]. It is uncertain as to whether the reported ethnic group was actually the baby's and not the mother's or whether it was in fact reported by the mother and not a health professional[31].

Socioeconomic circumstances was considered a potential mediator of the association between ethnicity and birth outcomes. It was assessed using the 2015 English Index of Multiple Deprivation (IMD)[32] for births in England and the 2014 Welsh IMD[33] for births in Wales. The IMD is a composite index of the relative deprivation of a defined geographical area, usually a homogeneous small area of relatively even size containing approximately 1,500 people. The index is computed from a series of indicators and statistics measuring the income, employment, health deprivation and disability, educational skills and training, housing and geographical access to services of the individuals living in an area. For the purpose of this analysis, areas were grouped into deciles of IMD, with the most deprived in the lowest decile and the least deprived in the highest decile. IMD was available for all births in the linked dataset.

An individual-level measure of socioeconomic circumstances, the National Statistics Socioeconomic Classification (NS-SEC), was available for a 10% sample of live births in which information on parental occupation recorded at birth registration was coded by the ONS. The NS-SEC is an occupationally based measure of employment relations and conditions of occupations, conceptually relevant in showing the structure of socioeconomic circumstances and explaining various social phenomena[34]. Individuals are grouped into nine categories, which are ordered starting with employment classes corresponding to better socioeconomic circumstances. To align with the direction of ordering of IMD, NS-SEC was reverse-coded so that the lowest category corresponded to the lowest socioeconomic classification and the highest category corresponded to the highest socioeconomic classification. A household NS-SEC was derived from parental occupation by assigning the higher parental NS-SEC classification to the household.

The linked dataset also included covariates such as mother's age, mother's country of birth which was grouped into UK and non-UK for this analysis, birth registration type (married parents, unmarried parents living at the same address, unmarried parents living at different addresses, or a sole registrant), year of birth between 2006 and 2012, and sex of child. These variables were included in our analyses as potential confounders of the association between socioeconomic circumstances measured using IMD and birth outcomes. Mother's age and year of birth were modelled as continuous variables and any departures from linear association with the outcomes were explored and fitted.

For peer review only - http://bmjopen.bmj.com/site/about/guidelines.xhtml

Analysis

The slope index of inequality (SII) and the relative index of inequality (RII) were estimated to describe the differences in birth outcomes across IMD[35,36]. The SII is a measure of absolute inequality which describes gradients in outcomes across subgroups with a natural ordering. It is expressed in terms of the difference between predicted outcomes of individuals in the highest versus lowest deprivation decile. The RII is a measure of relative inequality, expressed as the ratio of predicted outcome between the highest versus lowest level of deprivation. SII and RII were estimated using the method suggested in the WHO Handbook on Health Inequality Monitoring[37], which applied regression analysis – including appropriate modelling of any departures from linearity – to predict risks of birth outcomes in the most and least deprived individuals. Confidence intervals for the SII and RII were estimated by bootstrapping. As a sensitivity analysis, we repeated these analyses using household NS-SEC instead of IMD in the 10% sample of live births for which NS-SEC was available.

Two approaches were used to investigate the role of relative deprivation in the association between ethnic group and birth outcomes. First, the amount of variation in birth outcomes across ethnic groups which was explained by socioeconomic circumstances – measured using IMD deciles and modelled by linear regression – was estimated as the relative change in the residual ethnic group-level variance in outcomes as a function of relative deprivation[38,39]. The second approach, which assumed a causal link between ethnic group and IMD, and between IMD and adverse birth outcomes, involved structural equation modelling (SEM) to test an indirect effect through IMD in the effect of ethnicity on birth outcomes. This was based on the method described by Baron and Kenny[40] (Figure 1). It involved modelling the unadjusted effect of ethnic group on birth outcomes (path *c*, the *total effect,* not shown in Figure 1), and of ethnic group on IMD decile (path *a*), followed by estimation of the effect of ethnic group on birth outcomes (path *c'*) when adjusted for IMD (path *b*). Since the main exposure variable was multi-categorical, the *relative* indirect effect of IMD was estimated for each ethnic group, with white British ethnic group as the comparison group [41] followed by a Sobel test to assess the strength of evidence for an indirect effect[42]. Evidence of an indirect effect in at least one group is required to conclude overall mediation for the effect of the multi-categorical exposure[41].

The relative indirect effect, i.e. the proportion of total effect mediated, was calculated as *ab/c* or equivalently 1-(*c'*/*c*). A lack of evidence of effect in path *a* or *b* implied the absence of an indirect effect; a lack of evidence of effect in path *c* implied that there was no effect to be mediated. In either case, the relative indirect effect was meaningless, therefore not calculated. Linear, rather than logistic, regression was preferred for modelling all effects despite the birth outcomes being binary variables because explained variance measures in linear models are most intuitive,[43,44] as is the causal decomposition of bivariate associations in linear models, and given that the large number of observations in this linked

For peer review only - http://bmjopen.bmj.com/site/about/guidelines.xhtml

dataset, the effect estimates and standard errors from linear regression of binary outcomes are expected to be identical to those from logistic regression[45]. To correct for multiple testing, we used a stringent threshold of $p<0.001$ when assessing the evidence for direct and indirect effects. Goodness-of-fit of the SEM was tested using the root mean square error of approximation (RMSEA)[46], the comparative fit index (CFI)[47] and the Tucker-Lewis index (TLI)[48]. These indices range in value between 0 and 1. Smaller values of the RMSEA and larger values of both CFI and TLI represent better fit.

All analyses were of complete cases. Data management, manipulation and analyses were performed using Stata v13[49].

Patient and public involvement

A Patient and Public Involvement (PPI) group was formed to advise on the analysis plan, interpretation of results, and dissemination plans for this study. We formed the group by contacting organisations who would potentially be interested in this project and asking if they would like to join the PPI group. The organisations which sent a representative to our PPI meetings were: Bliss, the Race Equality Foundation, Sands, and the Lullaby Trust. In particular, the group provided guidance on ethnic groupings that were used in this study.

Results

Data on 4,744,666 infants were available in the linked dataset. We sequentially excluded 16,695 infants with gestational age of 43 weeks and above, 20,999 infants with missing birthweights and 72,040 infants with implausible birthweights. A total of 4,634,932 infants with complete data in most covariates were included in the main analysis and 463,533 in the sensitivity analysis (supplementary figure 1). There were 143 infants whose mothers' country of birth could not be coded as UK or non-UK, who were dropped from the adjusted models.

Table 1 is a summary of the characteristics of the infants included in the main analysis. The infants were largely of white British ethnic origin (65%) and mostly born to UK-born mothers (76%). The proportion of births in which the child's ethnicity was not stated was 6.2% overall, although it decreased from 9.9% in 2006 to 3.3% in 2012. There were 2.6% more male than female infants in the dataset. The number of births was distributed almost evenly over the 7 years covered by the dataset, although there was a gradual year-on-year increase in the number of births. The mean age of mothers was 29 years. Over half of registrations were by married parents and almost a third were by unmarried cohabiting parents. The rest were either joint registrations by parents living separately or single-parent registrants. There were

For peer review only - http://bmjopen.bmj.com/site/about/guidelines.xhtml
For peer review only

proportionately fewer infants in the less deprived IMD groups consistent with the distribution of the overall population across the deciles of IMD.

The overall proportion of preterm births was 5.6%. Infant and neonatal deaths were less common, occurring in 3.2 and 2.1 per 1,000 live births, respectively. However, more adverse birth outcomes occurred in infants born in deprived areas, overall (Table 2) and within ethnic group (Figure 2), and a disproportionate number of adverse outcomes occurred among ethnic minority infants (Table 2). Additionally, there were disproportionately more ethnic minority infants born in socio-economically deprived areas. In particular, there were higher proportions of black, Pakistani and Bangladeshi infants in the lower IMD deciles than the overall proportion of infants of these ethnicities, but a lower proportion of the same ethnicities in the higher IMD deciles; the opposite was true for white infants (Supplementary figure 2). The distribution of risks of adverse outcomes across ethnic groups in the 10% sample used for the sensitivity analysis was virtually identical to that in the overall sample (Supplementary table 1).

The slope and relative indices of inequality in IMD across birth outcomes are presented in Table 3. They show that infants born in the most socio-economically deprived areas had between 1.47 and 2.29 times the risk of adverse birth outcomes of those in the least deprived areas. The slope and relative indices of adverse birth outcomes across household NS-SEC were similar to those based on IMD: infants born in the most socio-economically deprived households had between 1.49 and 2.19 times the risk of adverse birth outcomes of those in the least deprived households (Supplementary table 2). Table 3 also shows that a small proportion, between 0.7% and 3.1%, of the overall variance in birth outcomes at ethnic group level was attributable to within-group differences. This implies that most of the variance in birth outcomes was due to between-group differences. Between 25.8% and 35.1% of the variance in outcomes at ethnic group level was explained by IMD in both crude and adjusted regression models.

There was strong evidence of an indirect effect of ethnic group on birth outcomes through socioeconomic circumstances (Sobel test p-values <0.001). Infants of all ethnic groups except white non-British infants had higher adjusted risks of neonatal and infant mortality than white British infants (Supplementary table 3). The same was true for preterm birth, except there was no evidence of a difference for Pakistani infants, and mixed/other infants had lower adjusted risk of preterm birth than white British infants. The relative indirect effects varied across ethnic groups and across outcomes, from -20.1% for infant mortality in white non-British infants to 78.2% for preterm birth in Black African infants, all relative to white British infants (Table 4). Given that the indirect effects were estimated relative to the white British group, negative indirect effect did not imply suppression as it would in absolute mediation models, but a lower indirect effect than the comparison group. The structural equation

For peer review only - http://bmjopen.bmj.com/site/about/guidelines.xhtml

models were a good fit for the data: in all models the RMSEA was <0.001 and both CFI and TLI were >0.999.

For peer review only

For peer review only - http://bmjopen.bmj.com/site/about/guidelines.xhtml

Table 1: Characteristics of the 4,634,932 infants born in England and Wales, 2006 – 2012

Ethnic group, n (%)	
White British	3,009,231 (64.9)
White (other)	340,526 (7.4)
Indian	132,651 (2.9)
Pakistani	180,269 (3.9)
Bangladeshi	62,948 (1.4)
Black Caribbean	47,505 (1.0)
Black African	154,076 (3.3)
Mixed or other ethnic group	419,970 (9.1)
Ethnic group not stated	287,756 (6.2)
Sex of infant, n (%)	
Male	2,377,766 (51.3)
Female	2,257,166 (48.7)
Year of birth, n (%)	
2006	631,705 (13.6)
2007	646,902 (14.0)
2008	663,918 (14.3)
2009	659,807 (14.2)
2010	671,265 (14.5)
2011	675,075 (14.6)
2012	686,260 (14.8)
Mother's age in years, mean (SD)	29.0 (6.0)
Mother's country of birth*, n (%)	
UK	3,507,324 (75.7)
Not UK	1,127,465 (24.3)
Birth registration type, n (%)	
Registration by married parents	2,499,063 (53.9)
Joint registration by unmarried parents (same address)	1,398,935 (30.2)
Joint registration by unmarried parents (different addresses)	450,500 (9.7)
Sole registrant	286,434 (6.2)
IMD decile, n (%)	
1 (most deprived)	662,767 (14.3)
2	598,259 (12.9)
3	541,795 (11.7)
4	489,932 (10.6)
5	439,270 (9.5)
6	422,908 (9.1)
7	388,665 (8.4)
8	382,585 (8.3)
9	366,851 (7.9)
10 (least deprived)	341,900 (7.4)
Preterm birth (<37 weeks gestation), n (%)	258,515 (5.6)
Neonatal mortality, n (per 1,000)	9,638 (2.1)
Infant mortality, n (per 1,000)	15,001 (3.2)

*Excludes 143 infants whose mother's country of birth is unclear or not stated

For peer review only - http://bmjopen.bmj.com/site/about/guidelines.xhtml

Table 2: Risks of adverse birth outcomes across ethnic groups and socioeconomic circumstances, England and Wales, births 2006 – 2012

	Risk of outcome		
Ethnic group	**Neonatal mortality per 1,000**	**Infant mortality per 1,000**	**Preterm birth %**
White British	1.8	2.9	5.5
White (other)	1.6	2.5	4.6
Indian	2.4	3.6	6.0
Pakistani	4.0	6.9	6.0
Bangladeshi	2.7	4.4	6.3
Black Caribbean	4.0	6.0	8.2
Black African	3.4	5.2	6.2
Mixed/other	2.1	3.4	5.6
Not stated	2.4	3.5	5.6
Index of multiple deprivation decile			
1 – most deprived	2.8	4.7	6.7
2	2.6	4.1	6.3
3	2.5	3.8	6.0
4	2.1	3.3	5.7
5	1.9	2.9	5.3
6	1.8	2.7	5.2
7	1.6	2.4	5.0
8	1.6	2.3	4.9
9	1.5	2.2	4.8
10 – least deprived	1.5	2.1	4.5
**Overall**	**2.1**	**3.2**	**55.8**

Risks are calculated as the number of events in each group divided by the number of infants in the group multiplied by 1,000 or 100

Table 3: Slope and relative indices of inequality (bootstrap 95% confidence intervals), and amount of variation in birth outcomes across ethnic groups explained by IMD, England and Wales, births 2006 – 2012

	**Neonatal mortality**	**Infant mortality**	**Preterm birth**
**Index of inequality across IMD**			
Slope index	1.48 (1.35 – 1.60)	2.66 (2.50 – 2.82)	2.15 (2.10 – 2.21)
Relative index	2.05 (1.92 – 2.18)	2.29 (2.17 – 2.41)	1.47 (1.46 – 1.49)
**Proportion of total variance:**			
within ethnic group (crude)	2.7%	3.1%	0.7%
explained by IMD (crude)	30.1%	34.0%	28.2%
explained by IMD (adjusted*)	25.8%	27.3%	35.1%

*adjusting for mother's age, mother's country of birth, registration type, year of birth and sex of child

For peer review only - http://bmjopen.bmj.com/site/about/guidelines.xhtml

Table 4: Relative indirect effect of ethnic group on birth outcomes, with 95% confidence intervals, England and Wales, births 2006 – 2012

	Neonatal mortality	Infant mortality	Preterm birth
White British	[comparison group]	[comparison group]	[comparison group]
White (other)	_	-20.1% (-35.9 to -4.4)	-8.8% (-9.5 to -8.0)
Indian	14.7% (8.5 to 20.1)	21.0% (10.4 to 31.6)	29.7% (19.9 to 39.4)
Pakistani	14.0% (11.3 to 16.7)	13.9% (12.2 to 15.6)	_
Bangladeshi	_	40.0% (24.4 to 55.2)	_
Black Caribbean	13.4% (10.5 to 16.3)	16.8% (13.0 to 20.7)	19.3% (17.0 to 21.5)
Black African	18.6% (14.1 to 23.1)	22.6% (18.5 to 27.0)	78.2% (64.2 to 92.2)
Mixed or other	_	_	_
Not stated	1.6% (0.8 to 2.3)	2.5% (0.6 to 4.4)	_

Indirect effects are differences relative to the comparison group; they were not calculated in groups and outcomes where there was no evidence of an unadjusted effect of ethnicity on socioeconomic circumstances (path *a*) or of an adjusted effect of socioeconomic circumstances on birth outcome (path *b'*)

Discussion

These data from a cohort of babies born in England and Wales between 2006 and 2012 showed poorer birth outcomes among infants of non-white minority ethnic groups. Specifically, ethnic minority infants born over this period had up to twice the risk of adverse birth outcomes of white British infants. These data also showed that among all ethnic groups there was evidence of a gradient in birth outcomes across levels of socioeconomic circumstances, with infants born in the most deprived areas experiencing between 1.47 and 2.29 times the risk of adverse birth outcomes compared with those born in the least deprived areas. Additionally, minority ethnic infants were more likely to be born in deprived areas. Our study aimed to explore the evidence for relative deprivation as the driving mechanism for differences in birth outcomes across ethnic groups. This was based on a causal model in which we hypothesised that relative deprivation was a consequence of minority ethnic status, which was then a cause of poorer birth outcomes.

We found that between a quarter and a third of the total between-ethnic group variance in birth outcomes was explained by relative deprivation. This finding, along with the evidence of the association between ethnicity and relative deprivation, and between relative deprivation and adverse birth outcomes, is consistent with a causal link between these factors, although not sufficient proof of causality. We also found strong evidence of an indirect effect through relative deprivation in the association between ethnic group and birth outcomes, with between 13.4% and 78.2% of the risk of adverse birth outcomes in non-white minority ethnic group infants estimated to be due to the higher levels of deprivation in these groups when compared to white British infants. Compared to white non-British infants relative deprivation mediated 8.8% to 20.1% of the excess risk of adverse birth outcomes in white British infants. Together, these findings support the hypothesis that relative deprivation is one mediating mechanism through which observed differences in birth outcomes across ethnic groups arise.

For peer review only - http://bmjopen.bmj.com/site/about/guidelines.xhtml

There may be other mediating mechanisms involving social, demographic or cultural factors, all of which are beyond the scope of this study, and this is further suggested by the finding of incomplete mediation across all groups and outcomes. The public health implication of these findings is that if we were to address the socioeconomic disadvantages borne by ethnic minorities in this population, we could reduce the between-ethnic group disparities in birth outcomes by between a quarter and a third in relative terms.

These conclusions are further supported by previous studies which highlight the associations between ethnicity, relative deprivation and birth outcomes. For example, other studies have shown that birth outcomes and health of infants and children tend to worsen with greater area-level poverty[50,51], lower household income[52] or in lower social classes[53,54], all of which are indicators of relative deprivation. Furthermore, previous studies in the UK[23,55] and these data also show a greater propensity for minority ethnic groups to live in socio-economically deprived areas and to suffer poorer outcomes. However, none of the studies we reviewed investigated the potential mediating effect of socioeconomic circumstances in the association between ethnicity and birth outcomes. Our study expands on previous literature by quantifying the extent to which socioeconomic circumstances contribute to ethnic disparities in birth outcomes.

We hypothesise that the relationship between ethnicity and socioeconomic circumstances, and between socioeconomic circumstances and adverse health outcomes are causal. A number of established findings support this hypothesis of causality and highlight possible mechanisms of effect. For example, language and cultural differences between minority and majority groups can create barriers to accessing or benefiting from information[56]. Minority groups are also more likely to experience various forms of discrimination based on their race, religion or culture, all of which may systematically disadvantage them in accessing services, housing and credit[57]; gaining employment[58], highlighted by the employment gap[59], and rising up the ranks while in employment[60]. In turn, poor socioeconomic circumstances can lead to poor health outcomes through: behavioural mechanisms e.g. tobacco, drug and alcohol use, poorer diet, lower utilisation of health services and contraception[9,61]; reduced access to material resources and safe environments resulting in greater mortality from pollution, poor housing and other health hazards; greater life stress caused by personal circumstances, leading to greater risk of depression, anxiety and poor mental health; with the cumulative effect of all these potential mechanisms over the life course on health and wellbeing within and across generations[62].

One limitation of this study is in the use of observational data to explore the causal relationships implied by the mediation model. Observational data are more prone to the occurrence of unmeasured confounding or reverse causality between the main exposure and the outcome, the mediator and the outcome, and between the exposure and the mediator. However, given that the exposure in this study

For peer review only - http://bmjopen.bmj.com/site/about/guidelines.xhtml

was ethnic group, there were unlikely to be any common causes or reverse causality between it and relative deprivation – the mediator in this analysis – and with birth outcomes. And although there was unlikely to be any reverse causality between relative deprivation and birth outcomes, this association was likely to be confounded by unmeasured factors since both factors may have had external common predictors. In our analysis, we adjusted this pathway for mother's age, mother's country of birth, birth registration type (a proxy measure for parental marital status), year of birth and sex of the child. Although this adjustment did not include factors such as education which may be common predictors of both relative deprivation and birth outcomes[63], the definition of IMD is broad and encompasses some of these factors, about which we did not have information in this dataset – a further limitation of the explanatory power of the study. We also did not have information about important parental factors such as smoking; therefore, there remains some potential for residual confounding. A criticism of the Baron and Kenny approach to the mediation analysis is that it has low statistical power[64]; however we had the benefit of a large number of observations which greatly improved our ability to detect associations. Another limitation of this study is the use of area level deprivation to model the socioeconomic circumstances of individuals; this was necessitated by the fact that individual level indicators of socioeconomic circumstances were not available for the whole sample. This has partly been addressed in the sensitivity analysis in which we have used family NS-SEC derived from parental NS-SEC as an alternative approach to measuring socioeconomic circumstances. Lastly, the ethnic group classifications used in this study may be of limited relevance to other settings.

These findings suggest that further reductions in adverse birth outcomes in England and Wales could be facilitated by interventions that reduce overall social inequality as this appears to be an important driver of variation in outcomes across ethnic groups. Such interventions must broaden their focus beyond health outcomes alone, and must aim to improve the totality of life circumstances among groups who experience the poorest outcomes.

For peer review only

For peer review only - http://bmjopen.bmj.com/site/about/guidelines.xhtml

Acknowledgements

We thank Bliss, the Race Equality Foundation, Sands, and the Lullaby Trust for their helpful involvement in the Patient and Public Involvement (PPI) consultation for this project. We also thank Alison MacFarlane, Hiranthi Jayaweera and Nirupa Dattani for their input during the planning and writing up of this study.

Author contributions

RG, JH, JJK, and MAQ conceived the study and were responsible for data acquisition. CO performed the statistical analysis with support from RG and MAQ. CO wrote the first draft, to which RG, JH, YL, JJK, and MAQ contributed and provided feedback during its development. All authors read and approved the final version of the manuscript.

Competing interests

All authors have completed the ICMJE uniform disclosure form at http://www.icmje.org/coi_disclosure.pdf and declare: no support from any organisation for the submitted work; no financial relationships with any organisations that might have an interest in the submitted work in the previous three years, no other relationships or activities that could appear to have influenced the submitted work.

Data availability

The data were provided by the Office for National Statistics (ONS) under a contractual agreement that does not permit the sharing of data. All requests for data access should be made directly to the ONS.

Ethical approval

The project was approved by the NRES Committee South Central - Oxford B, Ref 15/SC/0493.

Funding

This paper reports on an independent study which was funded by the NIHR Policy Research Programme in the Department of Health and Social Care, England (grant 108/0001). The Department of Health and

For peer review only - http://bmjopen.bmj.com/site/about/guidelines.xhtml

Social Care was not involved in any aspects of the study, and the views expressed are not necessarily those of the Department.

References

1. World Health Organization. Health across the life span. *World Heal Rep 1998*. 1998:61-111. http://www.who.int/whr/1998/en/whr98_ch3.pdf. Accessed March 8, 2018.

2. Black RE, Cousens S, Johnson HL, et al. Global, regional, and national causes of child mortality in 2008: a systematic analysis. *Lancet*. 2010;375(9730):1969-1987. doi:10.1016/S0140-6736(10)60549-1

3. Liu L, Johnson HL, Cousens S, et al. Global, regional, and national causes of child mortality: An updated systematic analysis for 2010 with time trends since 2000. *Lancet*. 2012;379:2151-2161.

4. Clift J, International Monetary Fund. *Health and Development : A Compilation of Articles from Finance & Development*. (Clift J, ed.). Washington; 2004. https://www.imf.org/external/pubs/ft/health/eng/hdwi/hdwi.pdf. Accessed March 8, 2018.

5. Norman P, Gregory I, Dorling D, Baker A. Geographical trends in infant mortality: England and Wales, 1970-2006. *Health Stat Q*. 2008;(40):18-29. https://search.proquest.com/docview/232834485/fulltextPDF/2F64288B1A974531PQ/1?accountid=13042. Accessed March 8, 2018.

6. Jakobsson M, Gissler M, Paavonen J, Tapper AM. The incidence of preterm deliveries decreases in Finland. *BJOG An Int J Obstet Gynaecol*. 2008;115(1):38-43. doi:10.1111/j.1471-0528.2007.01565.x

7. Schaaf J, Mol B, Abu-Hanna A, Ravelli A. Trends in preterm birth: singleton and multiple pregnancies in the Netherlands, 2000-2007. *BJOG An Int J Obstet Gynaecol*. 2011;118(10):1196-1204. doi:10.1111/j.1471-0528.2011.03010.x

8. Zeitlin J, Szamotulska K, Drewniak N, et al. Preterm birth time trends in Europe: A study of 19 countries. *BJOG An Int J Obstet Gynaecol*. 2013;120(11):1356-1365. doi:10.1111/1471-0528.12281

9. World Health Organisation. *Social Determinants of Health*. Second Edi. (Wilkinson R, Marmot M, eds.). Copenhagen; 2003.

10. Office for National Statistics. *Pregnancy and Ethnic Factors Influencing Births and Infant Mortality: 2013*. London; 2015. https://www.ons.gov.uk/peoplepopulationandcommunity/healthandsocialcare/causesofdeath/bulletins/pregnancyandethnicfactorsinfluencingbirthsandinfantmortality/2015-10-14.

11. The Marmot review. *The Marmot Review: Fair Society, Healthy Lives*. London; 2010. http://www.instituteofhealthequity.org/resources-reports/fair-society-healthy-lives-the-marmot-review/fair-society-healthy-lives-full-report-pdf.pdf. Accessed March 9, 2018.

12. Hollowell J, Kurinczuk JJ, Brocklehurst P, Gray R. Social and Ethnic Inequalities in Infant Mortality: A Perspective from the United Kingdom. *Semin Perinatol*. 2011;35(4):240-244. doi:10.1053/j.semperi.2011.02.021

13. Li Y, Quigley MA, Dattani N, et al. The contribution of gestational age, area deprivation and mother's country of birth to ethnic variations in infant mortality in England and Wales: A national cohort study using routinely collected data. *PLoS One*. 2018;13(4):e0195146.

For peer review only

For peer review only - http://bmjopen.bmj.com/site/about/guidelines.xhtml

doi:10.1371/journal.pone.0195146

14. Cangiano A. Net Migration as a Target for Migration Policies: A Review and Appraisal of the UK Experience. *Int Migr*. 2016;54(2):18-33. doi:10.1111/imig.12223

15. Coleman D. Immigration , Population and Ethnicity: The UK in International Perspective. *Migr Obs*. 2013:1-11. www.migrationobservatory.ox.ac.uk. Accessed March 13, 2018.

16. Zeitlin J, Mohangoo A, Delnord M. *European Perinatal Health Report: Health and Care of Pregnant Women and Babies in Europe in 2010*.; 2015. doi:10.1080/03639045.2017.1415927

17. McLaren E. Births by parents' country of birth, England and Wales: 2011. *Off Natl Stat*. 2012. https://www.ons.gov.uk/peoplepopulationandcommunity/birthsdeathsandmarriages/livebirths/bulletins/parentscountryofbirthenglandandwales/2012-08-30.

18. Coleman DA, Dubuc S. The fertility of ethnic minorities in the UK, 1960s-2006. *Popul Stud (NY)*. 2010;64(1):19-41. doi:10.1080/00324720903391201

19. Public Health England. Local action on health inequalities amongst ethnic minorities. Public Health Matters. https://publichealthmatters.blog.gov.uk/2018/08/06/local-action-on-health-inequalities-amongst-ethnic-minorities/. Published 2018. Accessed May 1, 2019.

20. Jeffries N, Zaslavsky AM, Diez Roux A V, et al. Methodological Approaches to Understanding Causes of Health Disparities. *Am J Public Health*. 2019;109(S1):S28-S33. doi:10.2105/AJPH.2018.304843

21. Nazroo JY. The structuring of ethnic inequalities in health: Economic position, racial discrimination, and racism. *Am J Public Health*. 2003;93(2):277-284. doi:10.2105/AJPH.93.2.277

22. Karlsen S, Nazroo JY. Relation between racial discrimination, social class, and health among ethnic minority groups. *Am J Public Health*. 2002;92(4):624-631. doi:10.2105/AJPH.92.4.624

23. Nazroo J, Karlsen S. Ethnic inequalities in health: social class, racism and identity. *Res Find*. 2001;10(10). http://www.lancaster.ac.uk/fass/projects/hvp/pdf/fd10.pdf. Accessed December 9, 2017.

24. Hilder L, Moser K, Dattani N, Macfarlane A. Pilot linkage of NHS Numbers for Babies data with birth registrations. *Heal Stat Q*. 2007;(33):25-33. https://pdfs.semanticscholar.org/128c/a2310d5d5028d5a5cc9a69d27dc3ac20f385.pdf. Accessed March 13, 2018.

25. Tukey J. *Exploratory Data Analysis*. Addison-Wesley; 1977. https://pdfs.semanticscholar.org/2f40/fd06bc9fd00a27437e14ed171e96e4fd9326.pdf. Accessed February 1, 2018.

26. World Health Organization. *International Statistical Classification of Diseases and Related Health Problems - 10th Revision (ICD10)*. WHO; 2010. http://apps.who.int/classifications/icd10/browse/Content/statichtml/ICD10Volume2_en_2010.pdf.

27. Moser K, Hilder L. Assessing quality of NHS Numbers for Babies data and providing gestational age statistics. *Health Stat Q*. 2008;37:15-23. http://researchonline.lshtm.ac.uk/24366/.

28. Office for National Statistics. *Ethnic Group Statistics: A Guide for the Collection and Classification of Ethnicity Data*. London; 2003. http://scholar.google.com/scholar?hl=en&btnG=Search&q=intitle:Ethnic+group+statistics+A+guide+for+the+collection+and+classification+of+ethnicity+data#1.

29. Kurinczuk JJ, Hollowell J, Brocklehurst P, Gray R. *Inequalities in Infant Mortality Project Briefing*

For peer review only

For peer review only - http://bmjopen.bmj.com/site/about/guidelines.xhtml

Paper 1. Infant Mortality: Overview and Context. Oxford; 2009. https://www.npeu.ox.ac.uk/downloads/files/infant-mortality/Infant-Mortality-Briefing-Paper-1.pdf.

30. Bosveld K, Connolly H, Rendall M. *A Guide to Comparing 1991 and 2001 Census Ethnic Group Data.* London; 2006. http://webarchive.nationalarchives.gov.uk/20151014015820/http://www.ons.gov.uk/ons/rel/ethnicity/focus-on-ethnicity-and-identity/a-guide-to-comparing-1991-and-2001-census-ethnic-group-data/comparing-1991-and-2001-census-ethnic-group-data.pdf.

31. Moser K, Stanfield K, Leon D. Birthweight and gestational age by ethnic group, England and Wales 2005: introducing new data on births. *Health Stat Q*. 2008;(39):22-31, 34-55. http://www.ncbi.nlm.nih.gov/pubmed/18810886. Accessed November 27, 2017.

32. Smith T, Noble M, Noble S, Wright G, McLennan D, Plunkett E. *The English Indices of Deprivation 2015 - Technical Report*.; 2015. doi:http://dx.doi.org/http://www.communities.gov.uk/publications/corporate/statistics/indices2010technicalreport

33. Welsh Government. Welsh Index of Multiple Deprivation (WIMD) 2014. *Off Natl Stat*. 2014:104. doi:10.1136/vr.h476

34. Office for National Statistics. The National Statistics Socio-Economic Classification. https://www.ons.gov.uk/methodology/classificationsandstandards/otherclassifications/thenationalstatisticssocioeconomicclassificationnssecrebasedonsoc2010. Published 2016. Accessed June 14, 2018.

35. Regidor E. Measures of health inequalities: part 2. *J Epidemiol Community Heal*. 2004;58(11):900-903. doi:10.1136/jech.2004.023036

36. Pamuk ER. Social-class inequality in infant mortality in England and Wales from 1921 to 1980. *Eur J Popul*. 1988;4(1):1-21. doi:10.1007/BF01797104

37. WHO. Handbook on health inequality monitoring: with a special focus on low- and middle-income countries. http://apps.who.int/iris/bitstream/10665/85345/1/9789241548632_eng.pdf. Accessed March 13, 2017.

38. LaHuis DM, Hartman MJ, Hakoyama S, Clark PC. Explained variance measures for multilevel models. *Organ Res Methods*. 2014;17(4):433-451. doi:10.1177/1094428114541701

39. Snijders T, Bosker R. Modeled variance in two-level models. *Sociol Methods Res*. 1994;22(3):342-363. doi:10.1177/0049124194022003004

40. Baron RM, Kenny DA. The moderator-mediator variable distinction in social psychological research: conceptual, strategic, and statistical considerations. *J Pers Soc Psychol*. 1986;51(6):1173-1182. doi:10.1037/0022-3514.51.6.1173

41. Hayes AF, Preacher KJ. Statistical mediation analysis with a multicategorical independent variable. *Br J Math Stat Psychol*. 2014;67:451-470. doi:10.1111/bmsp.12028

42. Sobel ME. Asymptotic Confidence Intervals for Indirect Effects in Structural Equation Models. *Sociol Methodol*. 1982;13:290. doi:10.2307/270723

43. Tjur T. Coefficients of Determination in Logistic Regression Models - A New Proposal: The Coefficient of Discrimination. *Am Stat*. 2009;634(4):366-372. doi:10.1198/tast.2009.08210doi.org/10.1198/tast.2009.08210

44. Mittlböck M, Schemper M. Explained variation for logistic regression. *Stat Med*.

For peer review only - http://bmjopen.bmj.com/site/about/guidelines.xhtml

1996;15(19):1987-1997. doi:10.1002/(SICI)1097-0258(19961015)15:19<1987::AID-SIM318>3.0.CO;2-9

45. Hellevik O. Linear versus logistic regression when the dependent variable is a dichotomy. *Qual Quant*. 2009;43(1):59-74. doi:10.1007/s11135-007-9077-3

46. Hu LT, Bentler PM. Cutoff criteria for fit indexes in covariance structure analysis: conventional criteria versus new alternatives. *Struct Equ Model*. 1999;6:1-55.

47. Bentler PM. Comparative fit indexes in structural models. *Psychol Bull*. 1990;107:238-246.

48. Tucker LR, Lewis C. A reliability coefficient for maximum likelihood factor analysis. *Psychometrika*. 1973;38:1-10.

49. StataCorp. *Stata 13*. College Station, TX: StataCorp LP; 2013.

50. DeFranco EA, Lian M, Muglia LJ, Schootman M. Area-level poverty and preterm birth risk: A population-based multilevel analysis. *BMC Public Health*. 2008;8(1):316. doi:10.1186/1471-2458-8-316

51. Bonet M, Smith LK, Pilkington H, Draper ES, Zeitlin J. Neighbourhood deprivation and very preterm birth in an English and French cohort. *BMC Pregnancy Childbirth*. 2013;13(1):97. doi:10.1186/1471-2393-13-97

52. Opatowski M, Blondel B, Khoshnood B, Saurel-Cubizolles MJ. New index of social deprivation during pregnancy: Results from a national study in France. *BMJ Open*. 2016;6(4):e009511. doi:10.1136/bmjopen-2015-009511

53. Bartley M, Blane D. Inequality and social class. In: Scambler G, ed. *Sociology as Applied to Medicine*. Elsevier; 2008.

54. Poeran J, Maas AFG, Birnie E, Denktas S, Steegers EAP, Bonsel GJ. Social deprivation and adverse perinatal outcomes among Western and non-Western pregnant women in a Dutch urban population. *Soc Sci Med*. 2013;83:42-49. doi:10.1016/j.socscimed.2013.02.008

55. Gray R, Headley J, Oakley L, Kurinczuk JJ, Brocklehurst P, Hollowell J. *Inequalities in Infant Mortality Project Briefing Paper 3. Towards an Understanding of Variations in Infant Mortality Rates between Different Ethnic Groups in England & Wales*. Oxford; 2009. https://www.npeu.ox.ac.uk/downloads/files/infant-mortality/Infant-Mortality-Briefing-Paper-3.pdf.

56. La Placa V, Corlyon J. Barriers to inclusion and successful engagement of parents in mainstream services: evidence and research. *J Child Serv*. 2014;9(3):220-234. doi:10.1108/JCS-05-2014-0027

57. Pager D, Shepherd H. The Sociology of Discrimination: Racial Discrimination in Employment, Housing, Credit, and Consumer Markets. *Annu Rev Sociol*. 2008;34(1):181-209. doi:10.1146/annurev.soc.33.040406.131740

58. Booth AL, Leigh A, Varganova E. Does ethnic discrimination vary across minority groups? Evidence from a field experiment. *Oxf Bull Econ Stat*. 2012;74(4):547-573. doi:10.1111/j.1468-0084.2011.00664.x

59. Department for Work & Pensions. Labour Market Status by Ethnic Group: data to September 2013. 2014;(January). https://www.gov.uk/government/uploads/system/uploads/attachment_data/file/269689/labour-market-status-by-ethnic-group.pdf. Accessed March 1, 2018.

60. Schiller B. *The Economics of Poverty and Discrimination*. 9th ed. Upper Saddle River, NJ: Pearson Prentice Hall; 2004.

For peer review only - http://bmjopen.bmj.com/site/about/guidelines.xhtml

61. Ellis A, Fry, R. Regional health inequalities in England. *Reg Trends*. 2010;42(1):60-79. doi:10.1057/rt.2010.5

62. Marmot M, Ryff CD, Bumpass LL, Shipley M, Marks NF. Social inequalities in health: Next questions and converging evidence. *Soc Sci Med*. 1997;44(6):901-910. doi:10.1016/S0277-9536(96)00194-3

63. Cutler D, Lleras-Muney A. *Education and Health: Evaluating Theories and Evidence*. Cambridge, MA; 2006. doi:10.3386/w12352

64. Hayes AF. Beyond Baron and Kenny: Statistical Mediation Analysis in the New Millennium. *Commun Monogr*. 2009;76(4):408-420. doi:10.1080/03637750903310360

For peer review only

For peer review only - http://bmjopen.bmj.com/site/about/guidelines.xhtml

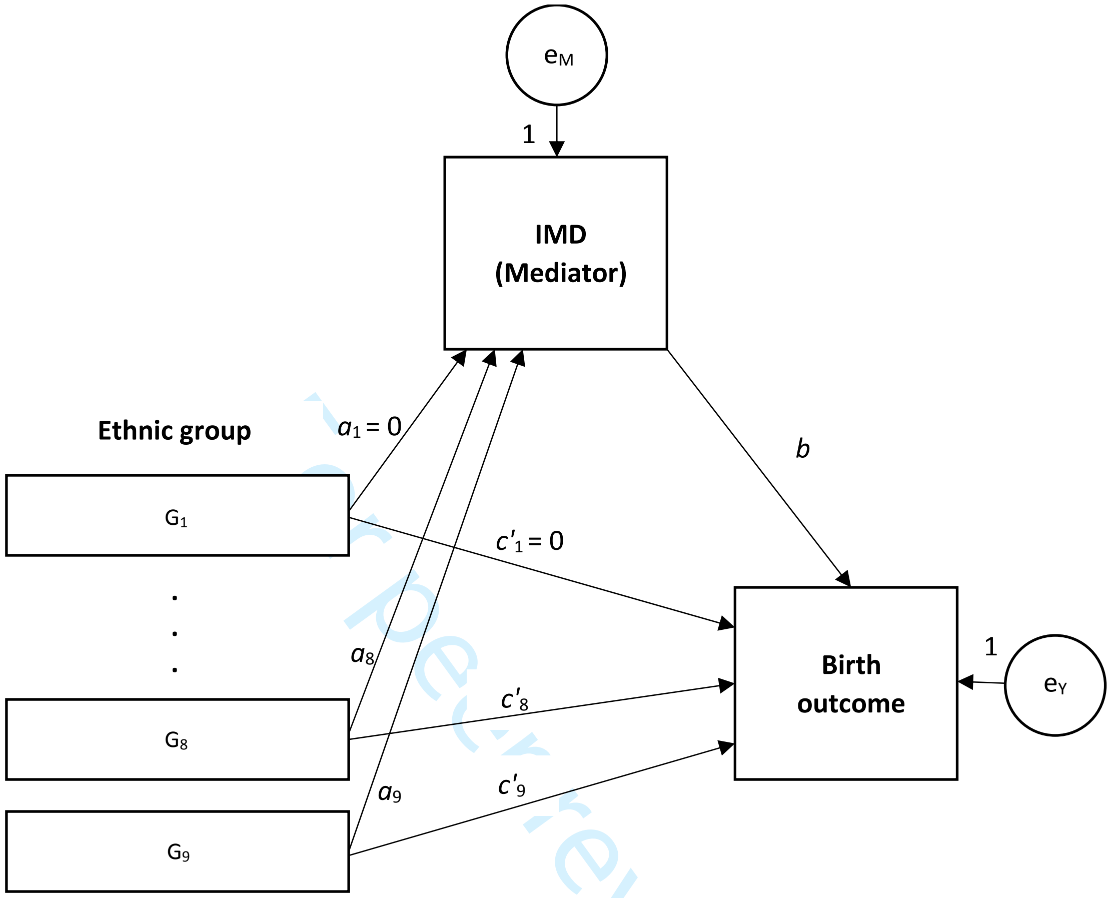

Figure 1: Mediation model for multi-categorical exposure variable

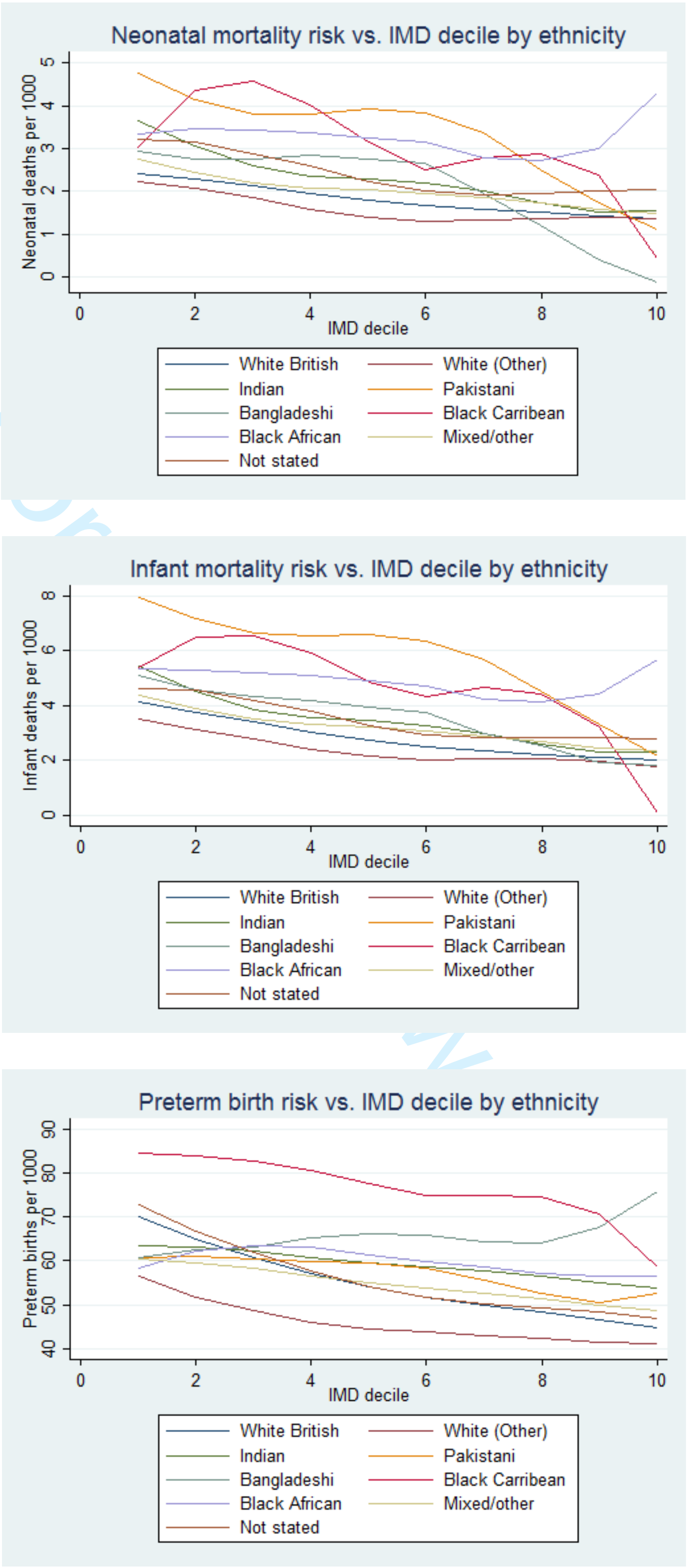

Figure 2: Distribution of neonatal mortality (top), infant mortality (centre) and preterm births (bottom) across IMD deciles within the ethnic groups, England and Wales, births 2006 – 2012

Supplementary material

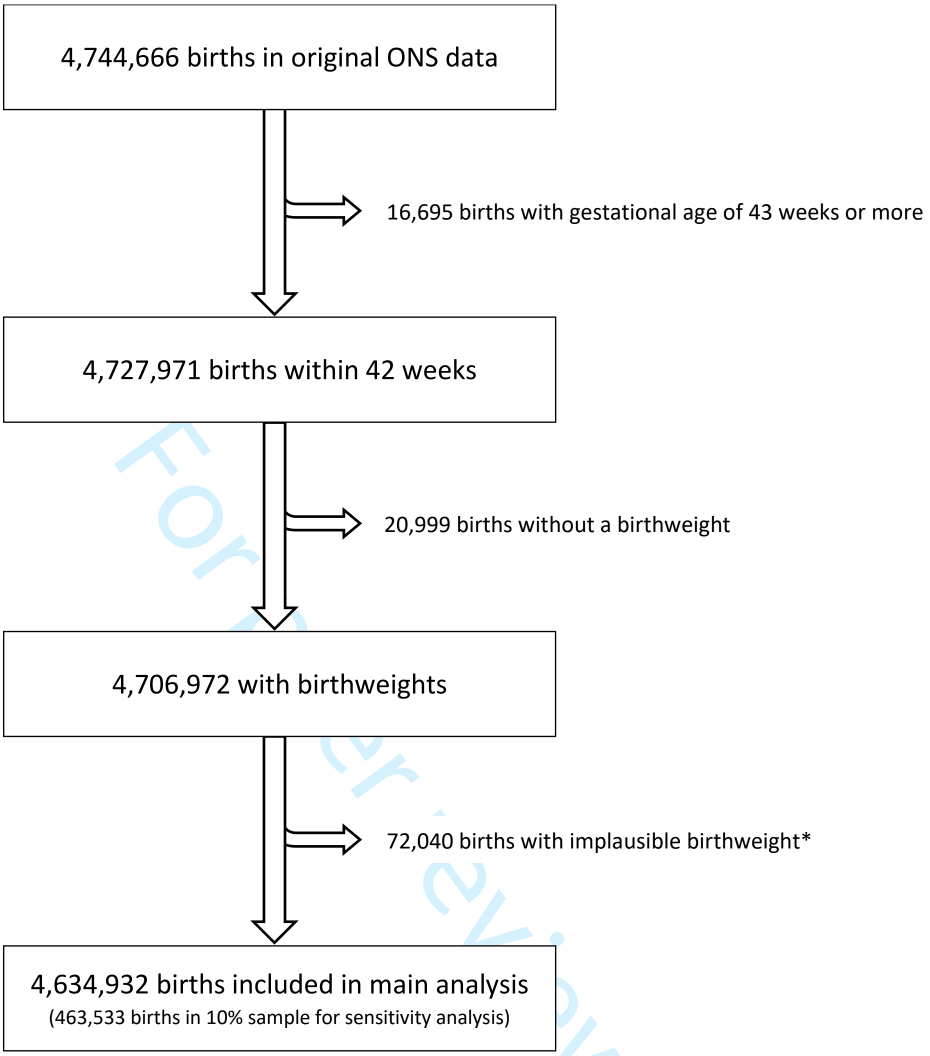

* A birthweight is considered implausible if it exceeds twice the interquartile range above and below the sex, gestation and ethnic group-specific median weight

Supplementary figure 1: Sample profile of the infants included in the analysis, England and Wales, births 2006 – 2012

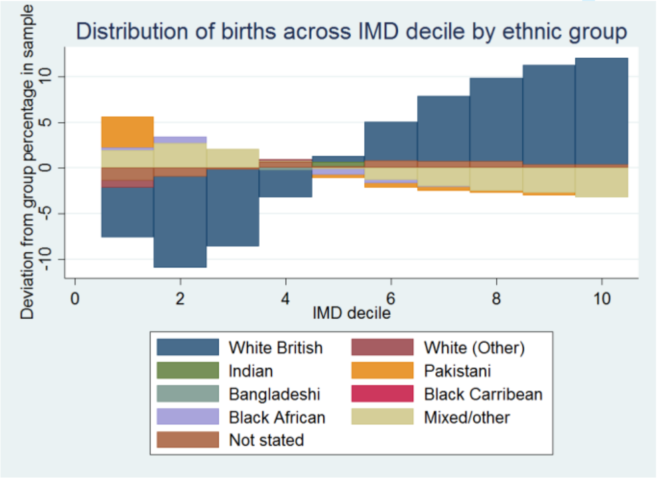

Supplementary figure 2: Distribution of births in each ethnic group and IMD relative to the ethnic group proportion of births, England and Wales, births 2006 – 2012

For peer review only - http://bmjopen.bmj.com/site/about/guidelines.xhtml

Supplementary table 1: Risks of adverse birth outcomes across ethnic groups and NS-SEC classification in the 10% sample of births, England and Wales, births 2006 – 2012

	Risk of outcome		
Ethnic group	**Neonatal mortality per 1,000**	**Infant mortality per 1,000**	**Preterm birth %**
White British	1.8	2.8	5.6
White (other)	1.7	2.4	4.7
Indian	2.6	3.8	5.6
Pakistani	4.0	6.9	6.0
Bangladeshi	2.4	4.4	5.9
Black Caribbean	3.9	6.4	8.2
Black African	4.1	5.8	6.3
Mixed/other	2.3	3.7	5.7
Not stated	2.5	3.8	5.5
NS-SEC classification			
9 never worked, unemployed	3.4	5.4	7.4
8 routine occupations	2.5	4.1	6.4
7 semi-routine occupations	2.6	4.3	6.2
6 lower supervisory	2.0	3.0	5.8
5 small employers	2.0	3.5	5.5
4 intermediate occupations	2.3	3.4	5.6
3 lower managerial	1.8	2.6	5.1
2 higher professional	1.8	2.4	4.7
1 large employers	1.5	2.1	4.7
**Overall**	**2.1**	**3.3**	**5.6**

Supplementary table 2: Slope and relative indices of inequality (with bootstrap 95% confidence intervals) in the 10% sample of births, England and Wales, births 2006 – 2012

**Index of inequality across NS-SEC**	**Neonatal mortality per 1,000**	**Infant mortality per 1,000**	**Preterm birth %**
Slope index	1.41 (0.97 – 1.85)	2.71 (2.14 – 3.28)	2.33 (2.08 – 2.57)
Relative index	1.86 (1.46 – 2.26)	2.19 (1.78 – 2.59)	1.49 (1.42 – 1.56)

For peer review only - http://bmjopen.bmj.com/site/about/guidelines.xhtml

Supplementary table 3: Differences in risks of adverse birth outcomes across ethnic groups, England and Wales, births 2006 – 2012

**Ethnic group**	**Difference in risk of outcome (95% CI)**					
	**Neonatal mortality per 1,000**		**Infant mortality per 1,000**		**Preterm birth %**	
	**Unadjusted**	**Adjusted**	**Unadjusted**	**Adjusted**	**Unadjusted**	**Adjusted**
White British	[comparison group]	[comparison group]	[comparison group]	[comparison group]	[comparison group]	[comparison group]
White (other)	-0.2 (-0.3 – -0.1)	-0.3 (-0.4 – -0.1)	-0.4 (-0.6 – -0.2)	-0.5 (-0.7 – -0.3)	-0.9 (-1.0 – -0.8)	-1.0 (-1.1 – -0.9)
Indian	0.5 (0.3 – 0.8)	0.4 (0.2 – 0.7)	0.7 (0.4 – 1.0)	0.6 (0.2 – 0.9)	0.5 (0.4 – 0.6)	0.3 (0.2 – 0.5)
Pakistani	2.2 (2.0 – 2.4)	1.9 (1.7 – 2.1)	4.0 (3.8 – 4.3)	3.5 (3.2 – 3.8)	0.5 (0.4 – 0.6)	-0.1 (-0.2 – 0.0)
Bangladeshi	0.9 (0.5 – 1.2)	0.5 (0.2 – 0.9)	1.5 (1.1 – 2.0)	0.9 (0.5 – 1.4)	0.8 (0.6 – 0.9)	0.2 (-0.0 – 0.3)
Black Caribbean	2.2 (1.7 –2.6)	1.9 (1.5 – 2.3)	3.1 (2.6 – 3.6)	2.6 (2.1 – 3.1)	2.7 (2.5 – 2.9)	2.2 (1.9 – 2.4)
Black African	1.5 (1.3 – 1.8)	1.2 (1.0 – 1.5)	2.3 (2.0 – 2.6)	1.8 (1.5 – 2.1)	0.6 (0.5 – 0.8)	0.1 (0.0 – 0.3)
Mixed/other	0.3 (0.2 – 0.4)	0.2 (0.0 – 0.3)	0.5 (0.4 – 0.7)	0.3 (0.1 – 0.5)	0.1 (0.0 – 0.2)	-0.1 (-0.2 – -0.1)
Not stated	0.6 (0.4 – 0.8)	0.6 (0.4 – 0.8)	0.7 (0.5 – 0.9)	0.6 (0.4 – 0.9)	0.1 (0.0 – 0.2)	0.1 (-0.0 – 0.2)

Unadjusted difference in risk of outcomes represent the total effect (path c) of ethnic group on birth outcomes; differences adjusted for socioeconomic circumstances represent the direct effect (path c')

For peer review only - http://bmjopen.bmj.com/site/about/guidelines.xhtml

STROBE Statement—Checklist of items that should be included in reports of ***cohort studies***

	Item No	Recommendation	Page No
**Title and abstract**	1	(*a*) Indicate the study's design with a commonly used term in the title or the abstract	1
		(*b*) Provide in the abstract an informative and balanced summary of what was done and what was found	2
**Introduction**			
Background/rationale	2	Explain the scientific background and rationale for the investigation being reported	4
Objectives	3	State specific objectives, including any prespecified hypotheses	4
**Methods**			
Study design	4	Present key elements of study design early in the paper	5
Setting	5	Describe the setting, locations, and relevant dates, including periods of recruitment, exposure, follow-up, and data collection	5
Participants	6	(*a*) Give the eligibility criteria, and the sources and methods of selection of participants. Describe methods of follow-up<br>(*b*) For matched studies, give matching criteria and number of exposed and unexposed	5
Variables	7	Clearly define all outcomes, exposures, predictors, potential confounders, and effect modifiers. Give diagnostic criteria, if applicable	5-6
Data sources/ measurement	8*	For each variable of interest, give sources of data and details of methods of assessment (measurement). Describe comparability of assessment methods if there is more than one group	5-6
Bias	9	Describe any efforts to address potential sources of bias	6
Study size	10	Explain how the study size was arrived at	N/A
Quantitative variables	11	Explain how quantitative variables were handled in the analyses. If applicable, describe which groupings were chosen and why	6
Statistical methods	12	(*a*) Describe all statistical methods, including those used to control for confounding<br>(*b*) Describe any methods used to examine subgroups and interactions<br>(*c*) Explain how missing data were addressed<br>(*d*) If applicable, explain how loss to follow-up was addressed<br>(*e*) Describe any sensitivity analyses	7-8
**Results**			
Participants	13*	(a) Report numbers of individuals at each stage of study—eg numbers potentially eligible, examined for eligibility, confirmed eligible, included in the study, completing follow-up, and analysed<br>(b) Give reasons for non-participation at each stage<br>(c) Consider use of a flow diagram	8
Descriptive data	14*	(a) Give characteristics of study participants (eg demographic, clinical, social) and information on exposures and potential confounders<br>(b) Indicate number of participants with missing data for each variable of interest<br>(c) Summarise follow-up time (eg, average and total amount)	9-10
Outcome data	15*	Report numbers of outcome events or summary measures over time	9-11

For peer review only

Main results	16	(*a*) Give unadjusted estimates and, if applicable, confounder-adjusted estimates and their precision (eg, 95% confidence interval). Make clear which confounders were adjusted for and why they were included<br>(*b*) Report category boundaries when continuous variables were categorized<br>(*c*) If relevant, consider translating estimates of relative risk into absolute risk for a meaningful time period	9-11
Other analyses	17	Report other analyses done—eg analyses of subgroups and interactions, and sensitivity analyses	9-11
**Discussion**			
Key results	18	Summarise key results with reference to study objectives	12
Limitations	19	Discuss limitations of the study, taking into account sources of potential bias or imprecision. Discuss both direction and magnitude of any potential bias	13-14
Interpretation	20	Give a cautious overall interpretation of results considering objectives, limitations, multiplicity of analyses, results from similar studies, and other relevant evidence	14
Generalisability	21	Discuss the generalisability (external validity) of the study results	14
**Other information**			
Funding	22	Give the source of funding and the role of the funders for the present study and, if applicable, for the original study on which the present article is based	15

*Give information separately for exposed and unexposed groups.

Note: An Explanation and Elaboration article discusses each checklist item and gives methodological background and published examples of transparent reporting. The STROBE checklist is best used in conjunction with this article (freely available on the Web sites of PLoS Medicine at http://www.plosmedicine.org/, Annals of Internal Medicine at http://www.annals.org/, and Epidemiology at http://www.epidem.com/). Information on the STROBE Initiative is available at http://www.strobe-statement.org.

For peer review only
